# A Bambusuril That Responds to Anion Binding in Its Absorption Spectrum

**DOI:** 10.1021/acs.joc.5c03154

**Published:** 2026-04-02

**Authors:** Marie Grunová, Jay-ar Bautista dela Cruz, Petr Slávik, Vladimír Šindelář

**Affiliations:** † Department of Chemistry, Faculty of Science, 37748 Masaryk University, Brno 625 00, Czech Republic; ‡ RECETOX, Faculty of Science, Masaryk University, Brno 625 00, Czech Republic

## Abstract

We report the design, synthesis, and characterization of **BU1**, the first bambusuril derivative bearing phenyl substituents directly attached to the glycoluril nitrogen atoms. The macrocycle **BU1**, decorated with *p*-nitrophenyl groups, exhibits distinct optical responses upon anion binding, enabling the evaluation of anion affinity by UV–Vis spectroscopy for the first time. Complementary ^1^H NMR titrations confirmed a 1:1 host–guest stoichiometry and yielded association constants consistent with the UV–Vis data. Despite the presence of electron-withdrawing *p*-nitrophenyl groups, **BU1** forms relatively weak complexes with halide and perchlorate anions. NMR experiments and molecular modeling suggest that direct *N*-aryl substitution induces cavity distortion and reduces flexibility, which likely accounts for the observed decrease in binding strength.

## Graphical abstract

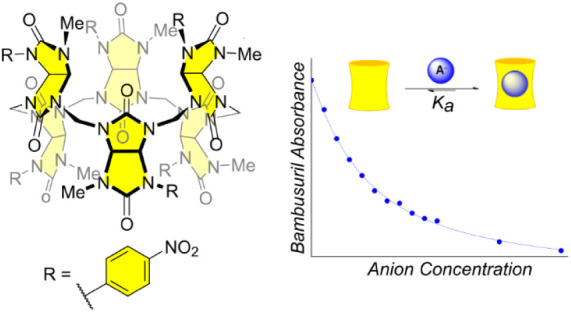

## Introduction

Anions are ubiquitous in nature and play critical roles across biological, industrial, and environmental systems.^1,2^ However, their diverse geometries, sizes, and charge distributions make anion-selective recognition significantly more challenging than that of cations. Consequently, the design of artificial receptors capable of binding anions with high affinity and selectivity has become a central objective in supramolecular chemistry.

Among various architectures developed for anion binding, hexameric bambusuril macrocycles have emerged as particularly powerful hosts due to their exceptional ability to encapsulate a wide range of anions in both aqueous and organic media.^3,4^ These macrocycles are synthesized via the condensation of 2,4-disubstituted (thio)­glycolurils with formaldehyde under acidic conditions.^5–8^ Numerous bambusuril derivatives have been reported, obtained either through the use of differently substituted glycoluril monomers or by postmacrocyclization modification.^9–12^ The relatively straightforward access to bambusurils bearing diverse substituents enables fine-tuning of their chemical and structural properties. For instance, we recently demonstrated that the anion-binding affinity of dodecabenzylbambusuril can be enhanced by incorporating electron-withdrawing groups onto its benzyl substituents.^13,14^ Increasing the electron-withdrawing character of these groups leads to a marked enhancement in binding strength. Similar principles have also been demonstrated on other macrocycles.^15,16^

Anion sensing represents an important application of such supramolecular host–guest systems.^1^ Optical supramolecular sensors employing absorption or emission spectroscopies are especially attractive due to their high sensitivity and low instrumental cost.^17–21^ To date, most bambusuril-based sensing studies have relied on NMR spectroscopy, which, while sensitive, is time-consuming and relatively expensive.^3^ UV–Vis spectroscopy has also been applied to quantify association constants of bambusuril-anion complexes by monitoring changes in the absorption spectra of either the anion or its counterion.^22,23^ However, no bambusuril derivative has yet been shown to exhibit a direct change in its own absorption spectrum upon anion binding. This limitation arises because the bambusuril framework is transparent above 210 nm. Moreover, bambusurils prepared to date feature chromophoric substituents that are distant from the anion-binding site and connected to the macrocyclic framework through flexible methylene bridges. This spatial and electronic separation likely prevents efficient coupling between anion encapsulation and optical response, explaining why the absorption spectra of these bambusurils remain unchanged upon guest inclusion.

Herein, we report the synthesis of **BU1**, the first bambusuril bearing *p*-nitrophenyl substituents directly attached to the glycoluril nitrogen atoms. The *p*-nitrophenyl groups were introduced through postsynthetic modification of the parent bambusuril, as direct macrocyclization of prefunctionalized glycolurils was found to be sterically disfavored. We describe the synthesis, anion-binding properties, and UV–Vis sensing behavior of **BU1**, demonstrating how direct chromophore conjugation to the macrocyclic core enables optical detection of anion inclusion and influences the binding affinity.

## Results and Discussion

Prior to the synthesis of **BU1** (Figure 1), we carried out semi-empirical PM6 calculations to assess the influence of the *p*-nitrophenyl substituents on the electron distribution within the macrocycle, particularly inside its cavity, which represents the anion-binding site. PM6 calculations were performed for **BU1**, and the results were compared with those of the previously studied macrocycle **BU2**, which features *p*-trifluoromethylthiobenzyl substituents^13,14^ (Figure 1). Our calculations showed that, despite possessing only six *p*-nitrophenyl substituents, the cavity of bambusuril **BU1** is more electropositive than that of **BU2**, which bears 12 electron-withdrawing *p*-trifluoromethylthiobenzyl substituents. These findings supported our expectation that **BU1** should function as a potent anion receptor, performing at least comparably to **BU2**.

**Figure 1:**
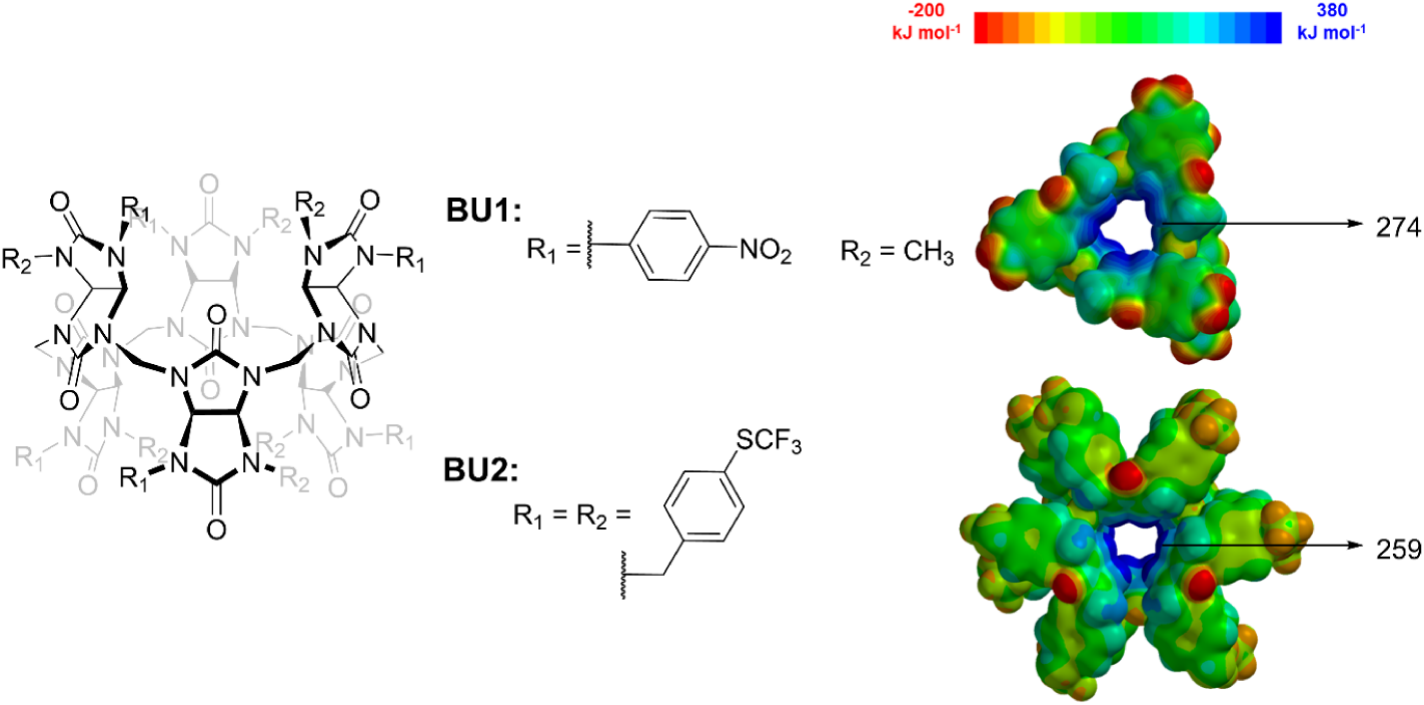
Electrostatic potential surfaces for BU derivatives **BU1** and **BU2**, with indicated cavity potentials.

Starting with the synthesis of **BU1**, we considered earlier reports describing unsuccessful efforts to prepare bambusuril derivatives bearing directly attached aryl groups. In particular, the acid-catalyzed condensation of 2,4-diphenylglycoluril with formaldehyde failed to afford the corresponding macrocycle, most likely due to steric repulsion between the phenyl substituents during macrocyclization.^9^ With this precedent in mind, we designed a glycoluril precursor **3** containing only one *p*-nitrophenyl group and one significantly less sterically demanding methyl substituent. The strongly electron-withdrawing nitrophenyl group was expected to decrease the electron density within the macrocyclic cavity, potentially enhancing anion-binding affinity, while the small methyl substituent should still enable macrocyclization of glycoluril **3**.

Glycoluril **3** was synthesized from glycoluril **1** (Scheme 1), whose preparation was recently reported.^12^ However, direct acid-catalyzed condensation of **3** with paraformaldehyde did not yield the desired macrocycle, confirming that even partial aryl substitution can hinder cyclization under standard conditions.

**Scheme 1:**
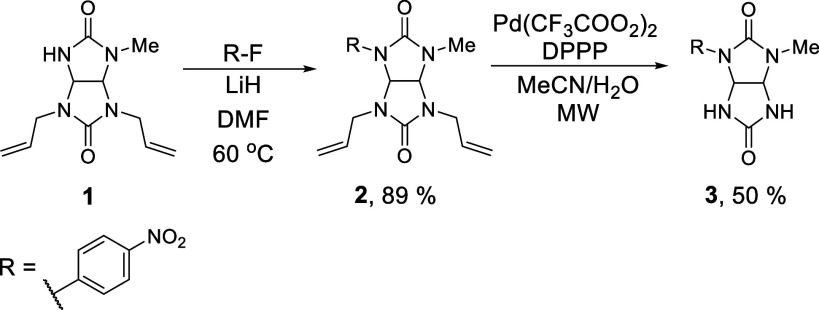
Stepwise Preparation of Glycolyril **3** from Glycoluril **1**

To overcome these limitations, we decided to prepare **BU1** by adapting our previously reported procedure, in which nitrogen atoms on already-made bambusuril could be deprotected, followed by postmacrocyclic modification.^12^ This modified route enabled the introduction of six *p*-nitrophenyl groups directly onto the macrocyclic framework, affording **BU1** (Scheme 2). The synthesis began with the preparation of asymmetric urea **5** from commercially available materials, followed by its condensation with 4,5-dihydroxyimidazolidin-2-one (DHI) to afford a mixture of diastereomers of glycolurils **6** and **7**. After separation, pure glycoluril **7** was obtained and subjected to acid-catalyzed macrocyclization to yield bambusuril **8**. Subsequent cleavage of the 1-phenylethyl protecting groups afforded the partially deprotected intermediate **9**. The target compound **BU1** was synthesized from **9** via an aromatic nucleophilic substitution reaction. Deprotonation of **9** with LiH in dry DMF generated the nucleophilic nitrogen centers, after which a DMF solution of 4-fluoronitrobenzene was added. The crude product contained residual anionic impurities, which were removed by washing with diethyl ether and water.

**Scheme 2:**
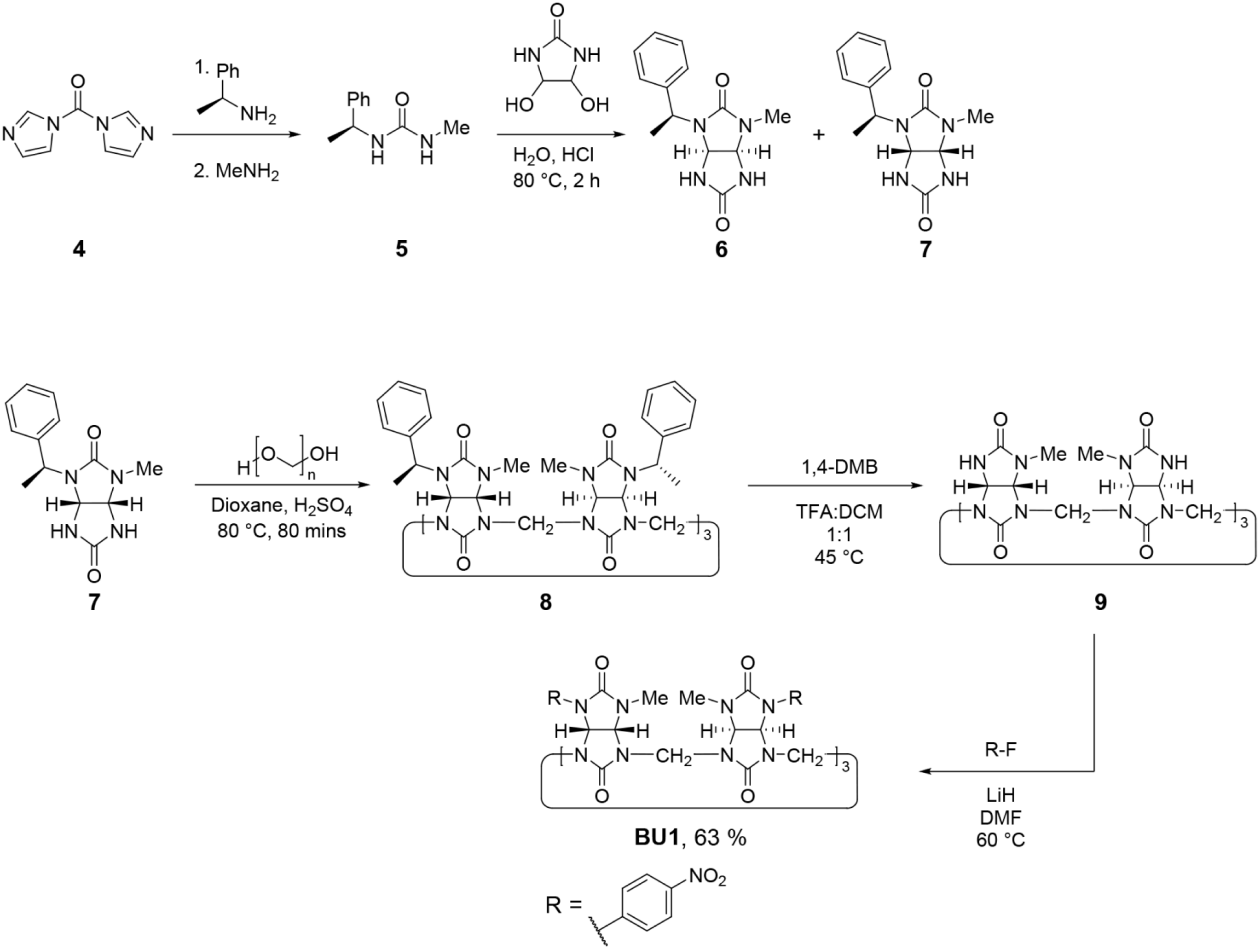
Preparation of **BU1** through Post-Macrocyclization Modification^a^

a The synthesis of **9** was previously reported.^12^

Host–guest properties of **BU1** and Cl^–^, Br^–^, and ClO_4_^–^, used as their tetrabutylammonium (TBA) salts in acetonitrile, were examined using UV–Vis spectroscopy. The absorption spectrum of **BU1** displayed a single band with a maximum at 324 nm, consistent with the presence of one type of chromophore in the molecule (Figure 2). Upon addition of an anion, the intensity of the absorption band decreased. The experimental data fitted well to a 1:1 binding isotherm, consistent with a single, well-defined host–guest equilibrium for complexes of 1:1 stoichiometry, and allowed calculation of association constant values given in Table 1. Because no shift or broadening of the absorption band was detected, the intensity decrease most likely arises from secondary effects accompanying anion inclusion rather than from a direct perturbation of the electronic transition. Possible contributing factors include slight geometric distortion at the *N*-aryl junctions, changes in the local dielectric environment within the cavity, or restricted motion of the aromatic substituents upon guest binding.

**Figure 2:**
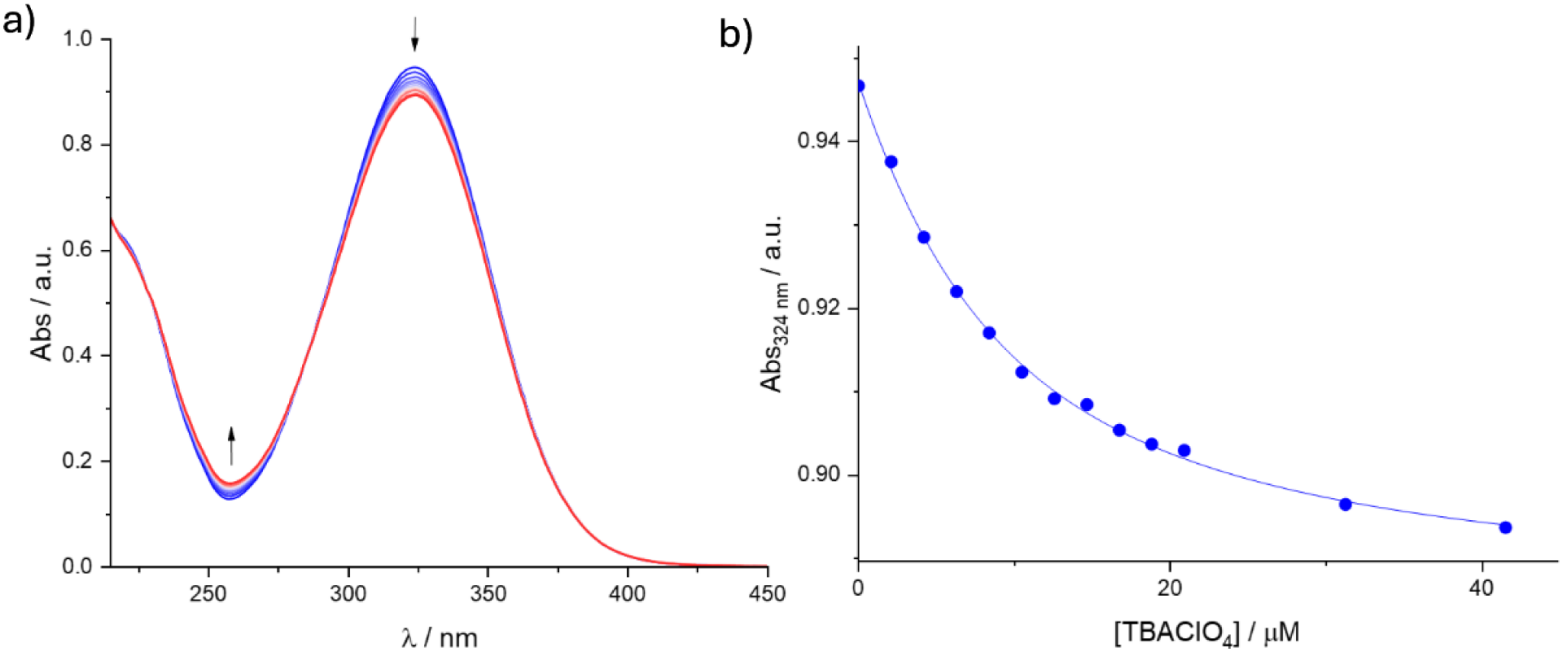
UV–Vis titration of **BU1** (10 μM) with TBAClO_4_ in MeCN. a) Changes in the UV–Vis spectra of **BU1** with an increasing amount of TBAClO_4_. b) Best fit of the 1:1 binding model to the change in absorbance of **BU1** at 324 nm with an increasing concentration of TBAClO_4_.

**Table 1:** Association Constants (*K*_a_) of **BU1** and **BU2** with Selected Anions Determined by UV–Vis and ^1^H NMR Titrations in Acetonitrile

	**BU1**		**BU2**
Anion	UV–Vis (M^–1^)	NMR (M^–1^)	NMR^a^ (M^–1^)
Cl^–^	(3.9 ± 0.4) × 10^3^	(4.0 ± 0.3) × 10^3^	(4.0 ± 2.3) × 10^10^
Br^–^	(3.8 ± 1.5) × 10^4^	(3.1 ± 0.3) × 10^4^	(6.3 ± 3.7) × 10^11^
I^–^	-	(7.8 ± 1.7) × 10^4^	(1.6 ± 0.9) × 10^12^
ClO_4_^–^	(1.3 ± 0.2) × 10^5^	(9.0 ± 1.0) × 10^4^	(3.8 ± 1.9) × 10^9^

a Taken from reference 14.

To validate the binding affinities of **BU1** toward anions obtained from UV–Vis titrations, the host–guest complexes were also studied by ^1^H NMR spectroscopy. Titrations were performed not only for Cl^–^, Br^–^, and ClO_4_^–^, but also for I^–^, which was not studied by UV–Vis due to the overlap of its absorption spectrum with that of **BU1**. Among these host–guest systems, ClO_4_^–^ produced the most distinct and well-defined spectral changes and is therefore discussed as a representative example. The remaining titrations, which exhibited similar patterns, are provided in the Supporting Information. Incremental addition of ClO_4_^–^ to a solution of **BU1** resulted in gradual and continuous shifts of all proton resonances (Figure 3a), consistent with a host–guest exchange process that is fast on the NMR time scale. The shifts reached saturation at approximately one equivalent of salt, indicating the formation of **BU1·**anion complex with a 1:1 stoichiometry. The largest chemical-shift changes were observed for signals *d*, *f*, and *g*. Notably, methine protons *c* and *d*, located inside the bambusuril cavity, responded differently: signal *d* shifted substantially, whereas signal *c* remained nearly unchanged. The aromatic protons (*a* and *b*) of the nitrophenyl substituents showed only minor variations. The experimental data for individual signals fitted well to the 1:1 binding isotherm (Figure 3b). Global analysis of all these binding isotherms was performed to determine association constants (Table 1) that are in good agreement with those obtained from UV–Vis titrations.

**Figure 3:**
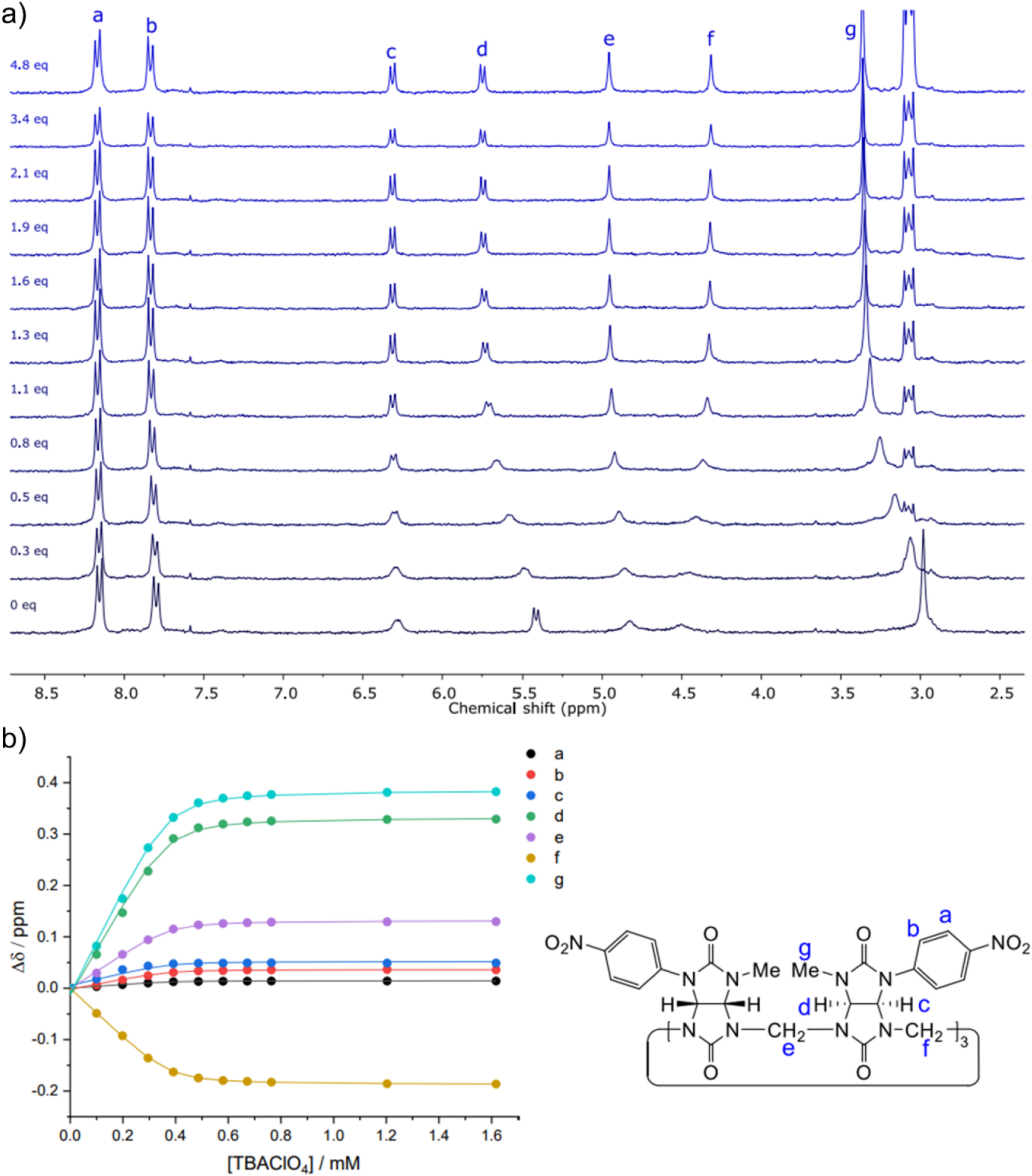
^1^H NMR (300.13 Hz, 25 °C, CD_3_CN) titration of **BU1** (0.40 mM) with TBAClO_4_. a) Best global fit of the 1:1 binding model to the shift of signals **a**–**g**. b) Changes in the chemical shifts of protons **a**–**g**.

The obtained association constants for the **BU1** complexes (Table 1) are significantly lower compared to bambusurils bearing benzyl substituents. For example, bambusuril **BU2** (Figure 1), bearing 12 benzyl substituents with SCF_3_ groups in *para*-position (Figure 1), binds each of the corresponding anions at least 6 orders of magnitude more strongly in the same solvent.^14^ Also, the selectivity of **BU2** is relatively low. This can be illustrated by the I^–^/Cl^–^ selectivity of 19.5 and 39.5 for **BU1** and **BU2,** respectively. The unexpectedly low stability of the **BU1·**anion complexes contrasts with our initial hypothesis that installing electron-deficient aromatic rings directly on the nitrogen atoms of **BU1** would enhance the anion affinity of the macrocycle. Based on the ^1^H NMR titration data, we attribute the lower binding to structural distortion of the **BU1** cavity induced by the phenyl substituents. This deformation is evidenced by the uneven chemical-shift changes of the bambusuril signals (*c–f*) upon anion addition, as discussed above. Such distortion likely disrupts the alignment of the six inward-facing C–H donors responsible for stabilizing the anion through CH···A^–^ interactions, thereby diminishing overall binding strength. Moreover, direct *N*-aryl substituents may increase the rigidity of the macrocyclic framework relative to benzyl substituents in other bambusurils, reducing the conformational flexibility required for optimal host–guest complementarity. Together, these effects plausibly account for the observed decrease in association constants.

Despite extensive efforts, single crystals of the bambusuril derivative **BU1** suitable for X-ray diffraction could not be obtained. To gain structural insight into the binding mode, we examined the geometry of **BU1** at the BLYP/def2-SVP level with D3(0) dispersion correction, using a CPCM acetonitrile model and starting from PM6-optimized coordinates (Figure 4A). After applying the dispersion correction, each of the two portals of **BU1** relaxes into a configuration in which two nitrophenyl substituents point outward from the cavity, while the remaining one is positioned at the portal entrance (Figure 4B). Because the aromatic groups are directly attached to the nitrogen atoms of the glycoluril building blocks, their relocation disrupts the idealized C3-symmetric portal arrangement typically observed in bambusurils bearing flexible N–CH_2_ substituents. Although each portal loses its individual symmetry upon distortion, the fact that both distort in the same manner ensures that the macrocycle as a whole retains its overall plane of symmetry. Moreover, the symmetric cavity typically found in bambusuril structures is significantly distorted. This steric restriction provides a structural rationale for the unexpectedly modest binding affinities, despite the presence of electron-withdrawing nitro groups that might otherwise be expected to enhance anion stabilization.

**Figure 4:**
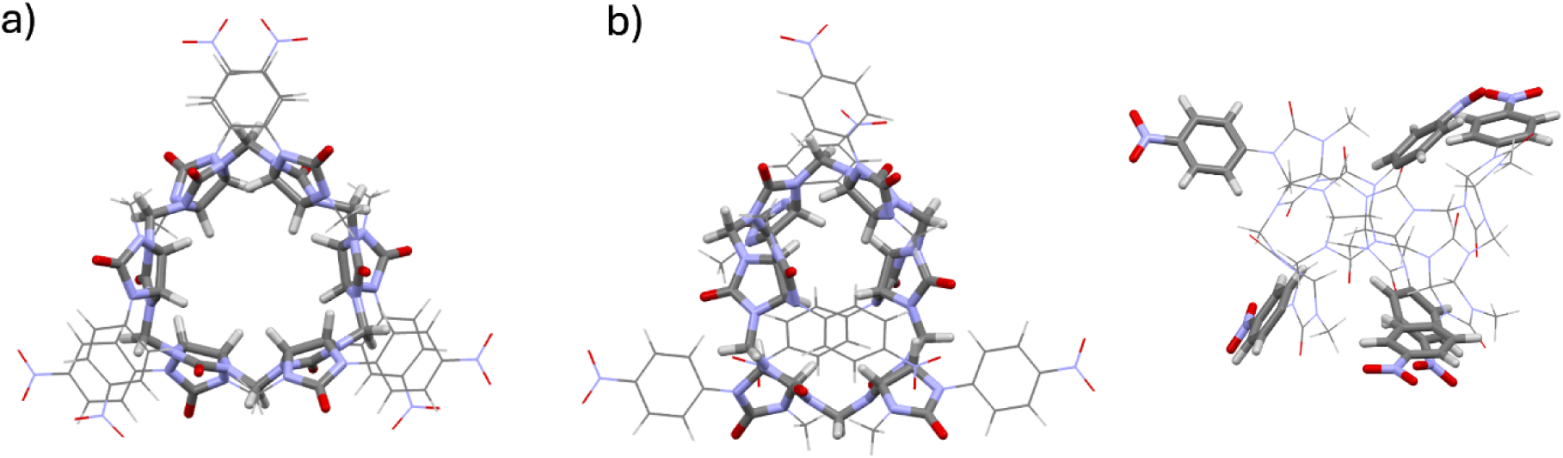
Optimized structures of **BU1**: a) PM6 geometry showing an idealized model. b) BLYP-D3(0)/def2-SVP geometry shown as top and side views.

## Conclusion

In summary, we have designed and synthesized the bambusuril derivative **BU1** with phenyl substituents directly attached to glycoluril constitutional units for the first time. The macrocycle **BU1**, functionalized with *p*-nitrophenyl groups, exhibits distinct changes in its absorption spectra upon anion addition, enabling the determination of association constants for corresponding host–guest complexes of 1:1 stoichiometry. The accuracy of the obtained *K*_a_ values was confirmed by complementary ^1^H NMR titrations, which also provided mechanistic insight into the influence of phenyl substitution on the supramolecular behavior of bambusurils. The obtained (sub)­millimolar affinities of **BU1** toward all investigated anions in acetonitrile are several orders of magnitude lower than those of bambusurils bearing benzyl substituents. This is in contrast to the electron-withdrawing nature of the *p*-nitrophenyl groups, which were expected to increase the macrocycle’s anion affinity by lowering the electron density in the central binding site. Molecular calculations indicate that the direct attachment of *p*-nitrophenyl substituents to nitrogen atoms distorts the macrocycle and reduces its flexibility, disrupting the cooperative C–H···A^–^ interactions that stabilize anions inside the **BU1** cavity. Thus, the structural effects outweigh the expected electronic benefit, leading to lower association constants. As this work paves the way toward bambusuril-based anion sensors, it also shows that the affinity of these macrocycles for anions can be tuned by installing bulky substituents on their portals.

## Experimental Section

### General

All reagents and solvents used were purchased from commercial suppliers and used without further purification. *trans*-4,5-Dihydroxyimidazolidin-2-one was synthesized based on a reported procedure.^24^ Compounds **1**, **5** to **9** were prepared according to literature methods.^12^ Reaction mixtures were heated on DrySyn heating blocks, and the reaction temperatures stated refer to the settings of the magnetic stirrer. Microwave syntheses were performed in pressurized vials using a CEM Discover SP microwave reactor. Reactions were monitored by thin-layer chromatography (TLC) using aluminum plates precoated with silica gel (60 F_254_, Merck) impregnated with a fluorescent indicator. TLC plates were visualized with ultraviolet light (λ = 254 nm) and by staining with aqueous potassium permanganate (KMnO_4_) or ceric ammonium molybdate (CAM), followed by heating. Flash column chromatography was performed using silica gel (60 Å, 40–63 μm, Fluorochem) or CombiFlash NextGen 300 from Teledyne ISCO. NMR spectra were recorded on Bruker Avance III HD 500 and Avance III 300 MHz spectrometers equipped with a BBFO probe with working frequency 500 or 300 MHz for ^1^H, 126 MHz for ^13^C­{^1^H}, and 471 or 282 MHz for ^19^F­{^1^H}. All experiments were recorded at 303.15 K. NMR chemical shifts (δ) are reported in parts per million (ppm) using residual solvent signals as references for the measured spectra in DMSO-*d*_6_ (^1^H = 2.50, ^13^C = 39.52) and CD_3_CN (^1^H = 1.94, ^13^C = 1.32). ^19^F NMR spectra were not referenced. Multiplicities are reported as singlet (s), doublet (d), doublet of doublets (dd), doublet of doublet of triplets (ddt), doublet of quartets (dq), triplet (t), quartet (q), multiplet (m), and broad (br). Signals were assigned with the aid of ^1^H–^1^H COSY, ^1^H^13^C HSQC, and ^1^H–^13^C HMBC experiments. High-resolution mass spectra (HRMS) were obtained on an Agilent 6224 Accurate-Mass Time-of-Flight (TOF) mass spectrometer. Samples were ionized by electrospray ionization (ESI) or atmospheric pressure chemical ionization (APCI). Matrix-assisted laser desorption ionization with detection by time-of-flight (MALDI-TOF) mass spectra were measured on the MALDI-TOF MS UltrafleXtreme (Bruker Daltonics). Samples were ionized by an Nd:YAG laser (355 nm) from a 2,5-dihydroxybenzoic acid (DHB) matrix. Melting points were measured on a Stuart SMP40 melting point apparatus. UV–Vis absorption spectra were recorded at 22 °C on a CARY 60 spectrometer from Agilent Technologies utilizing 1 cm quartz cuvettes.

### Glycoluril 2

Glycoluril **1** (195 mg; 0.83 mmol; 1.00 equiv) and LiH (33 mg; 4.09 mmol; 4.93 equiv) were transferred to a 50-mL round-bottom flask and purged with argon for 10 min. Afterward, DMF (2 mL; anhydrous) was added, producing a grayish white suspension, which was subsequently stirred at 60 °C for 30 min. In the meantime, *p*-NFB (184 mg; 1.30 mmol; 1.57 equiv) was dissolved in DMF (0.6 mL; anhydrous). This solution was added to the suspension dropwise over 5 min. The resulting reaction mixture changed color to bright green, then greenish-yellow and finally brownish-yellow. The reaction mixture was stirred at 60 °C with periodic monitoring by TLC. After 2.5 h, TLC indicated the disappearance of the starting glycoluril. Therefore, the heating was turned off, and instead, the reaction mixture was cooled with an ice/water bath. Residual lithium hydride was quenched with a saturated solution of NH_4_Cl (15 mL), with the observed evolution of gas. Furthermore, thick foam formed, which deflated after leaving the mixture in the fridge overnight. The product precipitated in the form of pale-yellow powder, which was isolated by filtration and subsequent washing with water (3 × 10 mL) and Et_2_O (1 × 5 mL) (263 mg, 89%. ^1^H NMR (500 MHz, DMSO-*d*_6_): δ 8.24 (d, *J* = 9.2 Hz, 2H), 7.77 (d, *J* = 9.2 Hz, 2H), 6.33 (d, *J* = 8.4 Hz, 1H), 5.84 (ddd, *J* = 11.3, 9.8, 5.1 Hz, 1H), 5.55 – 5.41 (m, 1H), 5.35 (d, *J* = 8.3 Hz, 1H), 5.29 – 5.17 (m, 2H), 4.96 – 4.81 (m, 2H), 4.15 – 4.01 (m, 1H), 3.96 – 3.76 (m, 2H), 3.52 – 3.44 (m, 1H), 2.90 (s, 3H). ^13^C­{^1^H} NMR (126 MHz, DMSO): δ 157.4, 155.3, 144.6, 142.6, 133.8, 133.2, 124.4, 120.4, 117.1, 116.4, 69.2, 67.0, 45.2, 44.9, 30.1. HRMS (APCl+) *m*/*z*: [M + H]^+^: Calcd for C_17_H_20_N_5_O_4_: 358.1510; found: 358.1507

### Glycoluril 3

Palladium­(II) trifluoroacetate (10 mg, 0.03 mmol, 0.11 equiv) and DPPP (15 mg, 0.04 mmol, 0.14 equiv) were mixed in a 4-mL vial and flushed with argon. Upon the addition of water (0.24 mL) and MeCN (0.38 mL), the obtained black suspension was stirred for 10 min at room temperature. Glycoluril **2** (100 mg, 0.28 mmol, 1.00 equiv) was transferred to a 35-mL microwave tube, purged with argon, and MeCN (0.38 mL) was added. The previously prepared solution of the palladium catalyst was transferred to the tube, and additional MeCN (0.38 mL) was added. The reaction was conducted in the microwave reactor with the following parameters: 120 °C, 200 W max, 45 min, 300 PSI max, medium stirring. The formation of the product was verified by TLC. The reaction mixture was filtered through a cotton ball, and the greenish-yellow filtrate was then concentrated on a rotary evaporator to give a dark yellowish solid. The crude mixture was separated by column chromatography using automatic flash chromatography. The product was isolated as a yellowish oil which solidified to give a pale-yellow powder (39 mg, 50%. ^1^H NMR (500 MHz, DMSO-*d*_6_): δ 8.19 (d, *J* = 9.3 Hz, 2H), 8.03 (s, 1H), 7.89 (s, 1H), 7.85 (d, *J* = 9.3 Hz, 2H), 5.98 (dd, *J* = 8.2, 2.5 Hz, 1H), 5.35 (dd, *J* = 8.2, 1.7 Hz, 1H), 2.81 (s, 3H). ^13^C­{^1^H} NMR (126 MHz, DMSO): δ 160.8, 154.3, 144.7, 141.3, 124.6, 117.0, 66.5, 65.7, 27.7. HRMS (APCl+) *m*/*z*: [M + H]^+^: Calcd for C_11_H_12_N_5_O_4_: 278.0884; found: 278.0883

### Bambusuril BU1

Bambusuril **9** (102 mg; 0.10 mmol; 1.00 equiv) and LiH (22 mg; 2.75 mmol; 27.54 equiv) were transferred to a 10-mL round-bottom flask, purged with argon, and DMF (0.5 mL; anhydrous) was added. The obtained white suspension was stirred at 60 °C for 30 min. *p*-NFB (125.7 mg; 0.89 mmol; 8.91 equiv) was dissolved in DMF (0.4 mL; anhydrous), producing a light yellow solution, which was subsequently added to the reaction mixture via syringe over 10 min. The change of color from white, through bright yellow, to dark yellowish green was observed. The reaction mixture was left stirring for 5 h, after which the formation of the product was confirmed by NMR spectroscopy. Afterward, the reaction mixture was cooled with an ice/water bath and milli-Q water was added (0.1 mL). This led to the formation of thick and stable foam, which gradually disappeared overnight with constant stirring. Attempting the removal of solvents with the foam present through evaporation under reduced pressure did not work well. Once the foam decreased significantly, the crude product was obtained by precipitation with Et_2_O and subsequent filtration. The obtained yellow fine powder was washed with diethyl ether (3 × 2.5 mL) and water (3 × 5 mL). Drying the product *in vacuo* gave the final anion-free macrocycle (111 mg, 63%. ^1^H NMR (500 MHz, DMSO-*d*_6_): δ 8.37 – 7.03 (m, 4H), 6.41 (s, 1H), 5.47 (s, 1H), 5.21 – 3.99 (m, 2H), 2.93 (s, 3H). ^13^C­{^1^H} NMR (126 MHz, DMSO): δ 158.2, 155.3, 144.0, 142.7, 124.2, 121.3, 69.8, 68.1, 52.5, 29.6. HRMS (ESI−) *m*/*z*: [M + Cl]^−^ Calcd for C_72_H_66_N_29_O_22_Cl: 1769.4554; found: 1769.4560, fragmentation due to nitro groups.

## Supplementary Material



## Data Availability

The data underlying this study are available in the published article and its online Supporting Information.

## Notes

^1^H and ^13^C­{^1^H} NMR spectra of new compounds and ^1^H NMR titration experiments (PDF)
