# Supplementary material for: A Bambusuril That Responds to Anion Binding in Its Absorption Spectrum
Source: J Org Chem. 2026 Apr 2;91(15):5298–304. doi: 10.1021/acs.joc.5c03154 (PMC13097242; doi:10.1021/acs.joc.5c03154)
Supplement: Supplementary file 1 [file jo5c03154_si_001.pdf]

Supporting Information

A Bambusuril That Responds to Anion Binding in Its Absorption Spectrum

Marie Grunová, Jay-ar Bautista dela Cruz, Petr Slávik, Vladimír Šindelář*

Department of Chemistry, Faculty of Science, Masaryk University, 625 00 Brno, Czech Republic; RECETOX, Faculty of Science, Masaryk University, 625 00 Brno, Czech Republic

Table of Contents

1. NMR Spectra S2
2. NMR Titrations Experiments S21
3. Computational Details S24

1. NMR Spectra

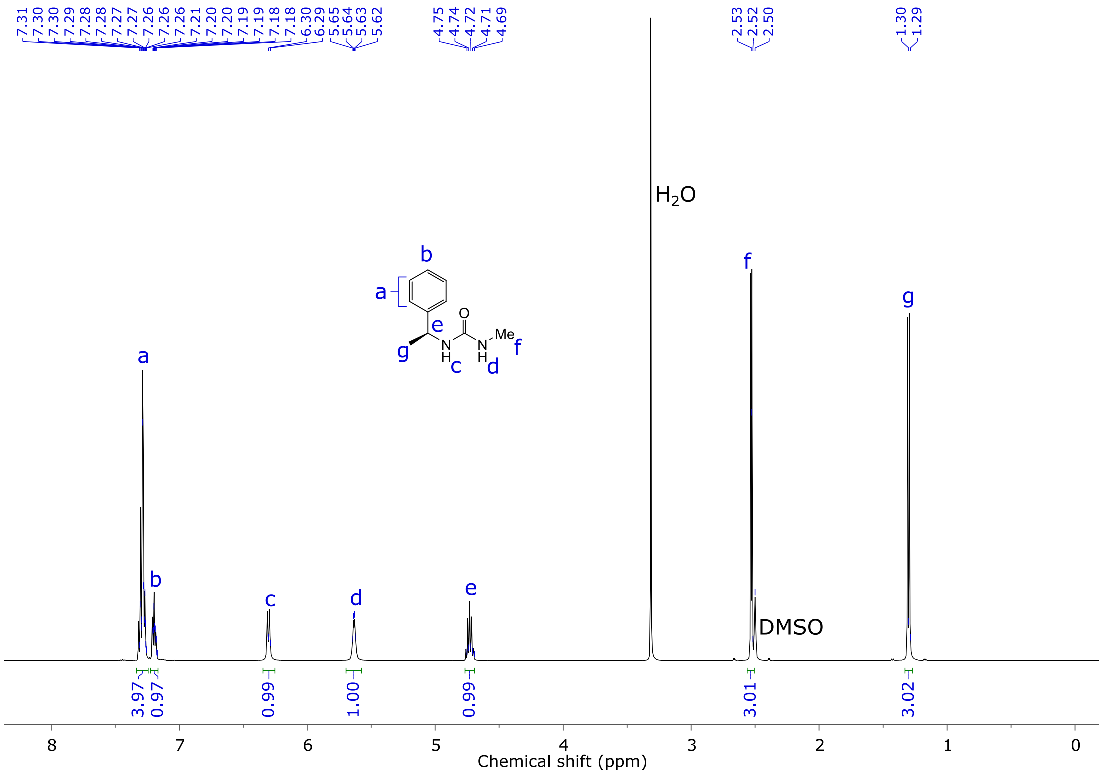

Figure S1: ^{1}H NMR spectrum (500.13 MHz, DMSO-d_6, 30 °C) of **5** (assignment).

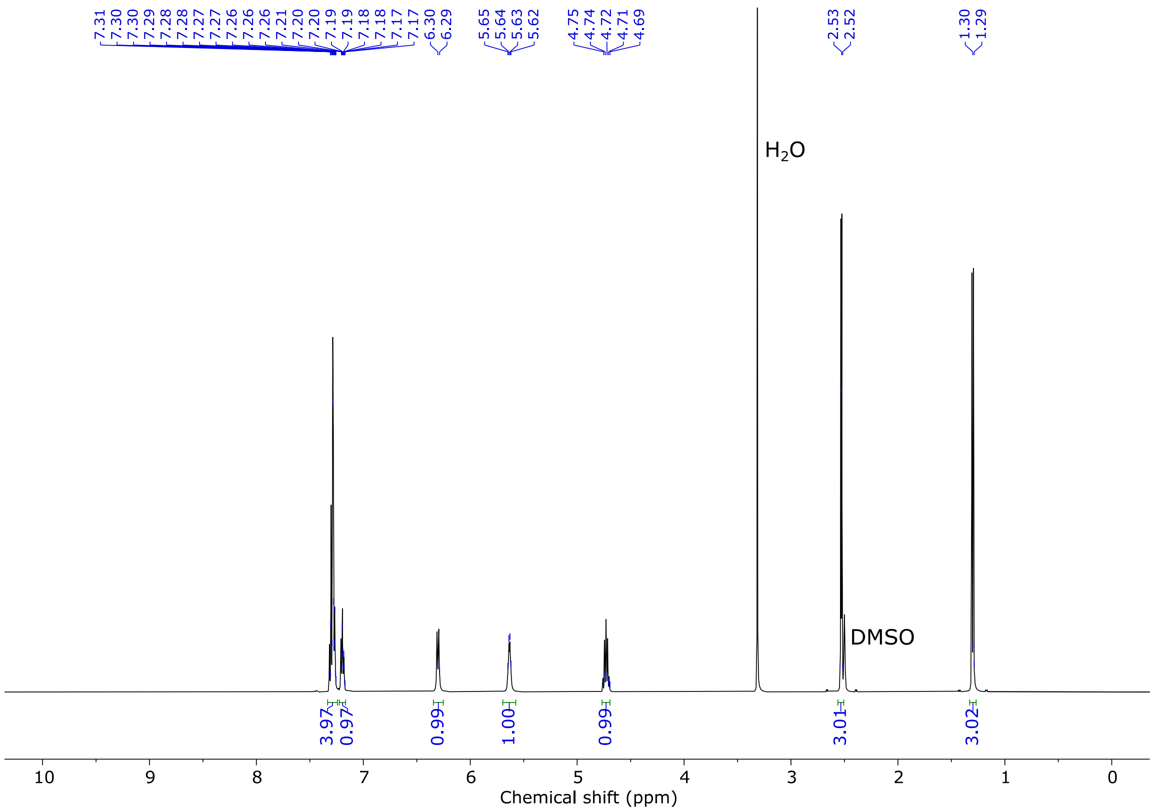

Figure S2: ^{1}H NMR spectrum (500.13 MHz, DMSO-d_6, 30 °C) of **5**.

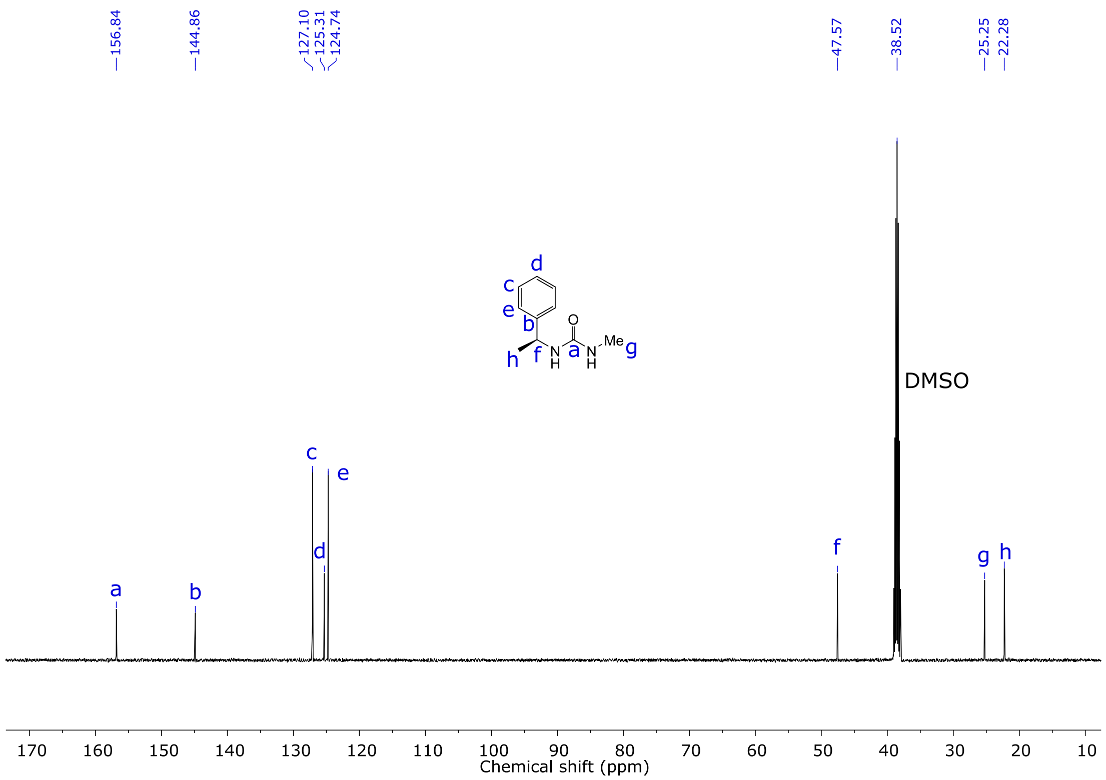

Figure S3: $^{13}C\{^1H\}$ NMR spectrum (125.77 MHz, DMSO-d_6, 30 °C) of **5** (assignment).

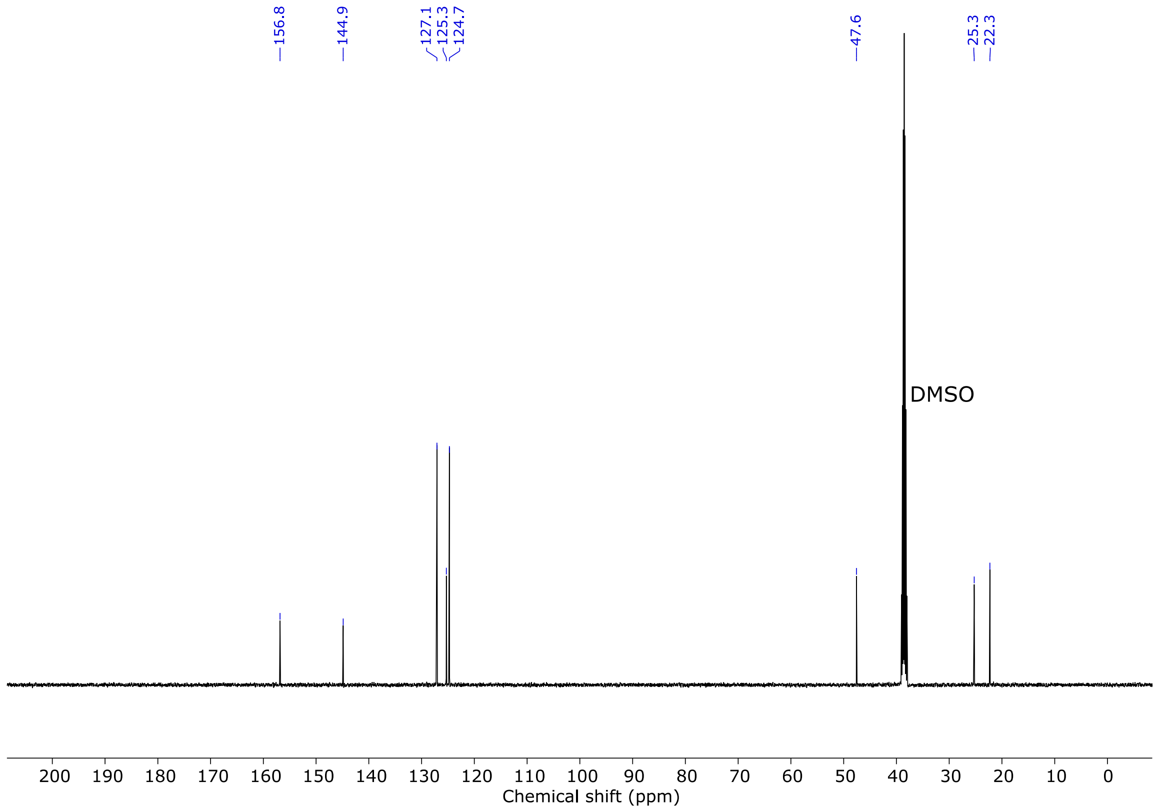

Figure S4: $^{13}C\{^1H\}$ NMR spectrum (125.77 MHz, DMSO-d_6, 30 °C) of **5**.

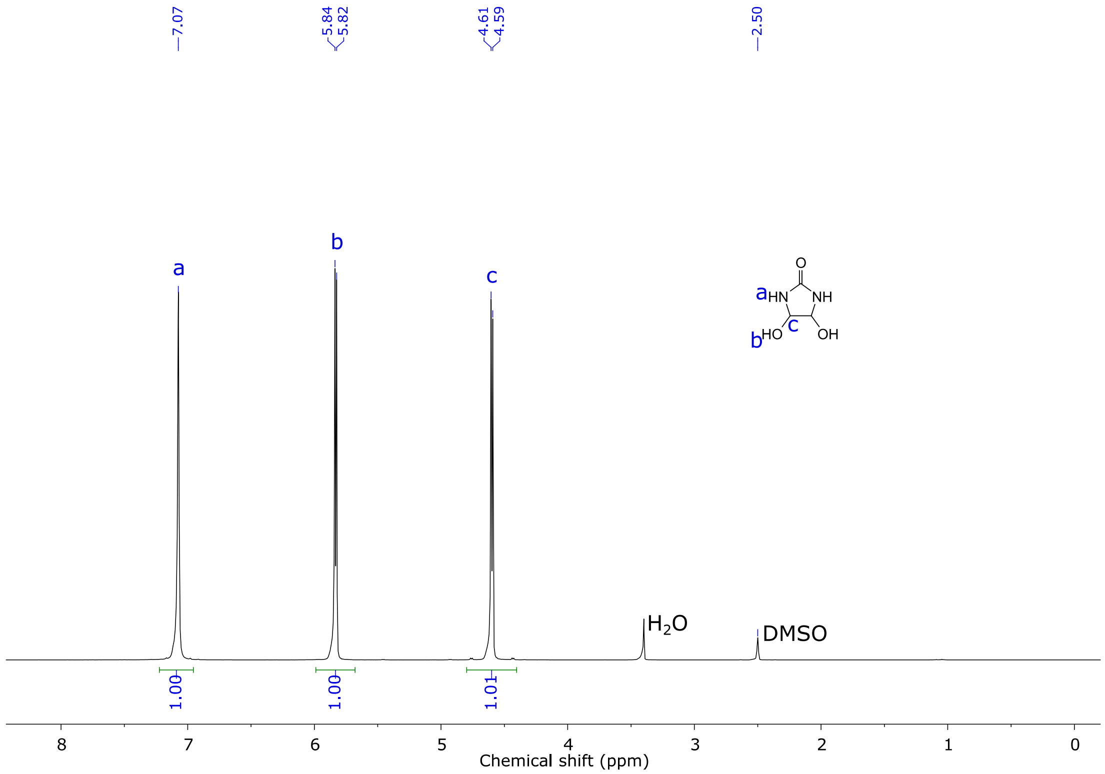

Figure S5: ^{1}H NMR spectrum (500.13 MHz, DMSO-d_6, 30 °C) of **DHI** (assignment).

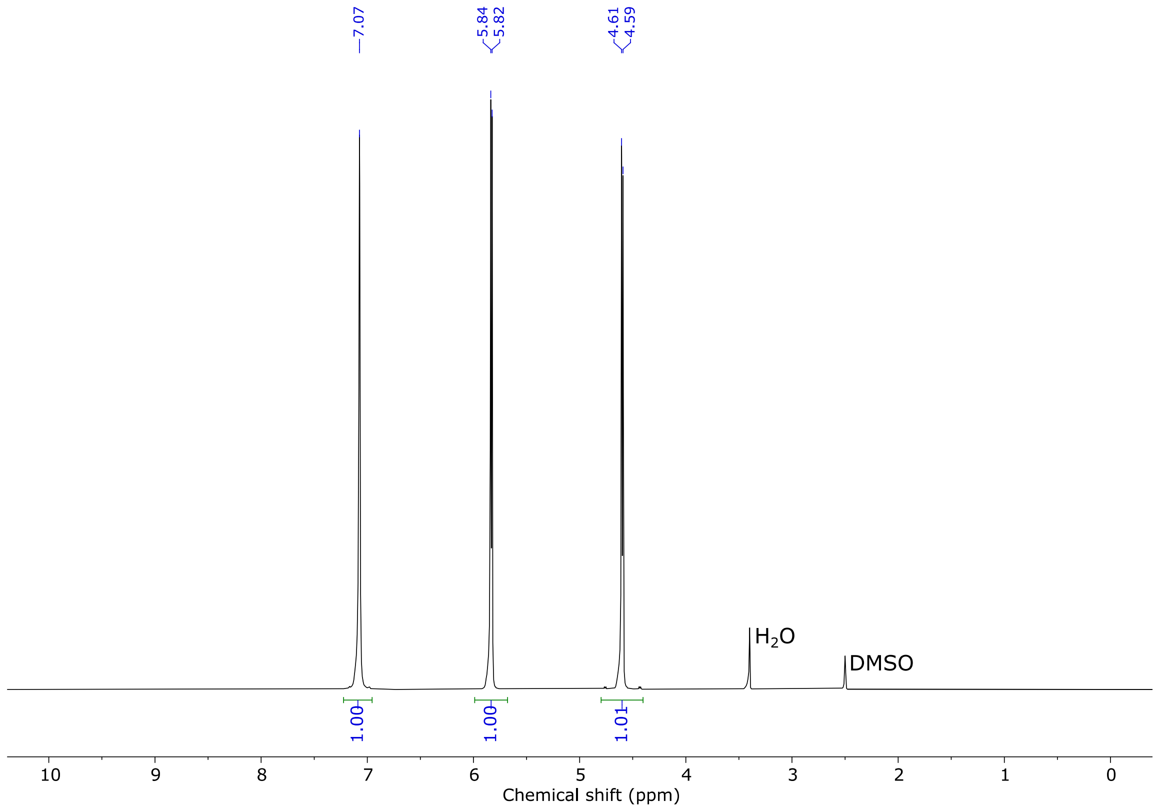

Figure S6: ^{1}H NMR spectrum (500.13 MHz, DMSO-d_6, 30 °C) of **DHI**.

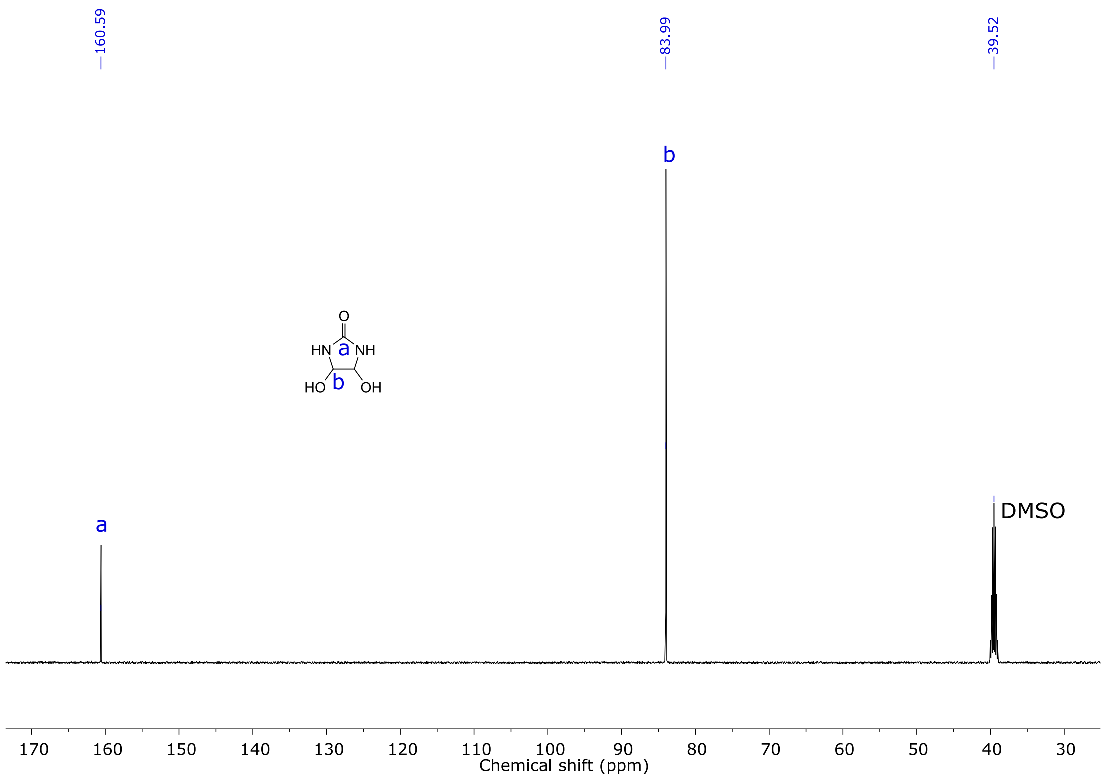

Figure S7: $^{13}C\{^1H\}$ NMR spectrum (125.77 MHz, DMSO-d_6, 30 °C) of **DHI** (assignment).

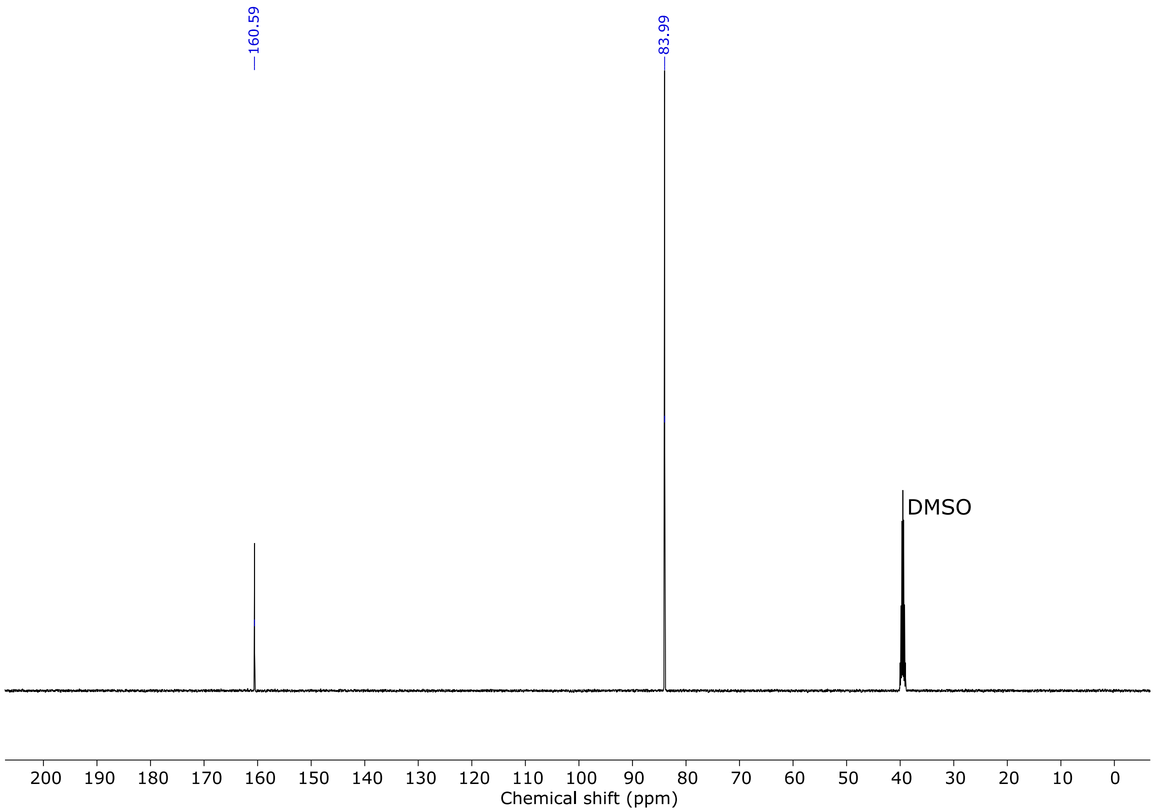

Figure S8: $^{13}C\{^1H\}$ NMR spectrum (125.77 MHz, DMSO-d_6, 30 °C) of **DHI**.

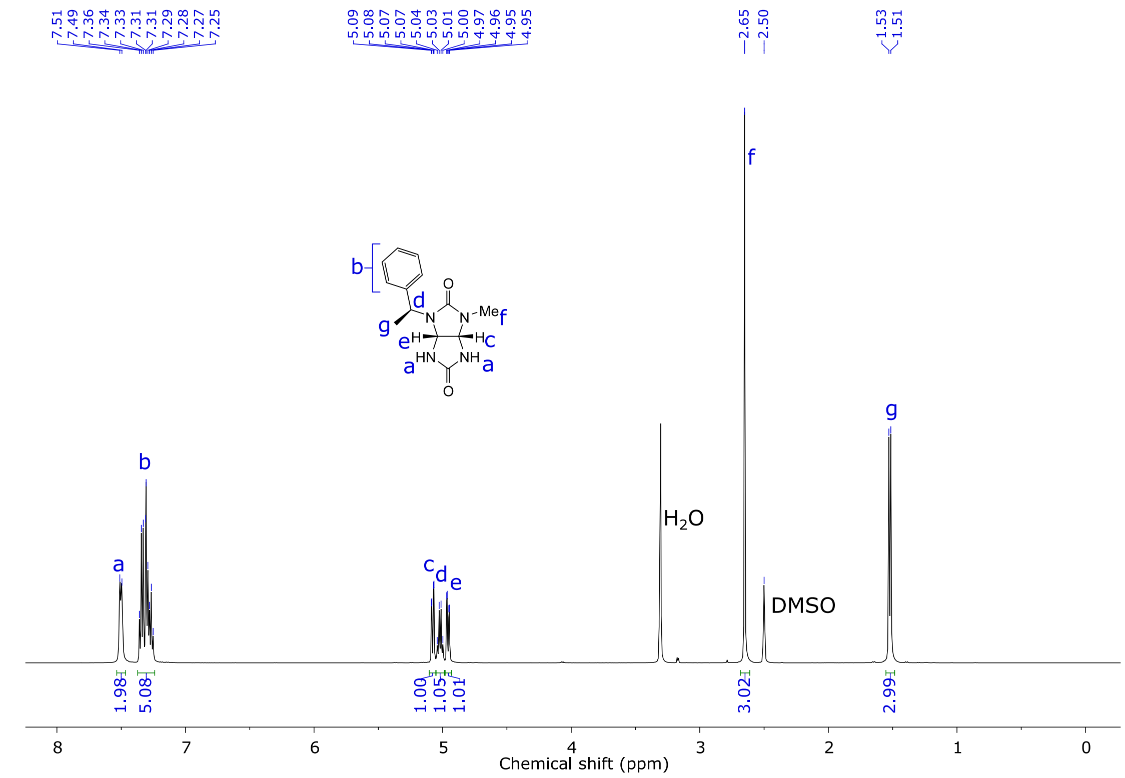

Figure S9: ^{1}H NMR spectrum (500.13 MHz, DMSO-d_6, 30 °C) of **6** (assignment).

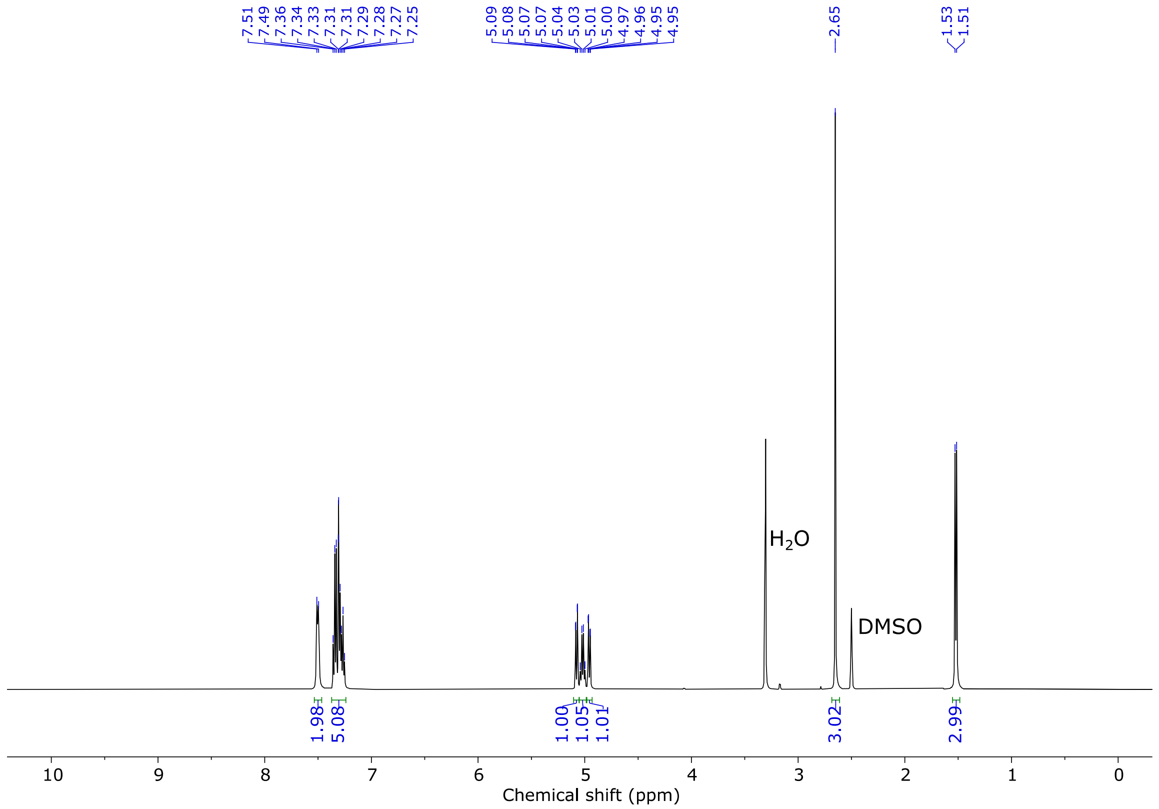

Figure S10: ^{1}H NMR spectrum (500.13 MHz, DMSO-d_6, 30 °C) of **6**.

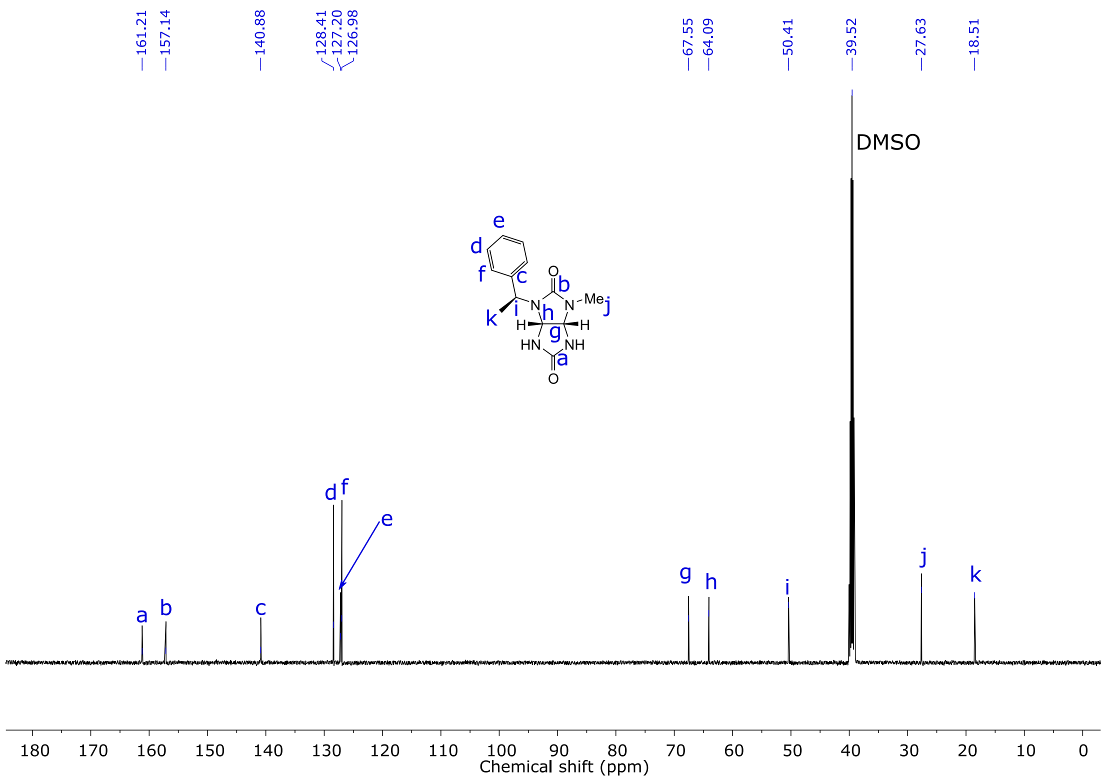

Figure S11: $^{13}C\{^1H\}$ NMR spectrum (125.77 MHz, DMSO-d_6, 30 °C) of **6** (assignment).

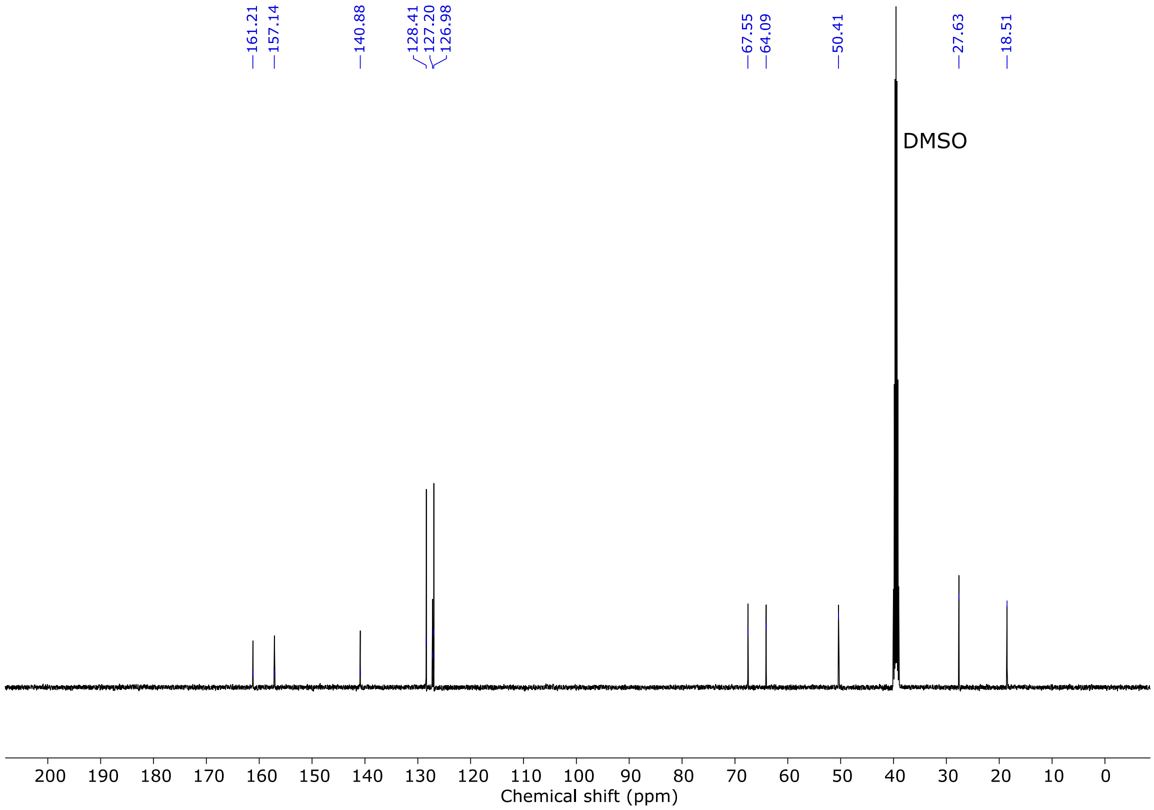

Figure S12: $^{13}C\{^1H\}$ NMR spectrum (125.77 MHz, DMSO-d_6, 30 °C) of **6**.

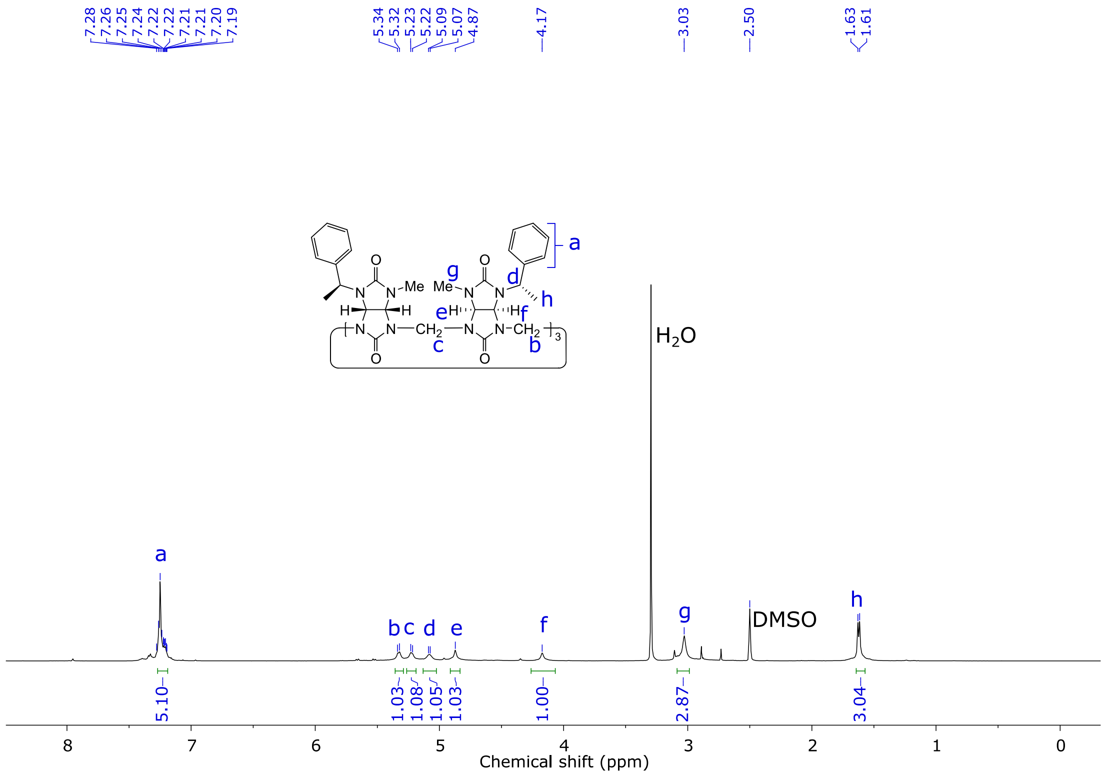

Figure S13: ^{1}H NMR spectrum (500.13 MHz, DMSO-d_6, 30 °C) of **8** (assignment).

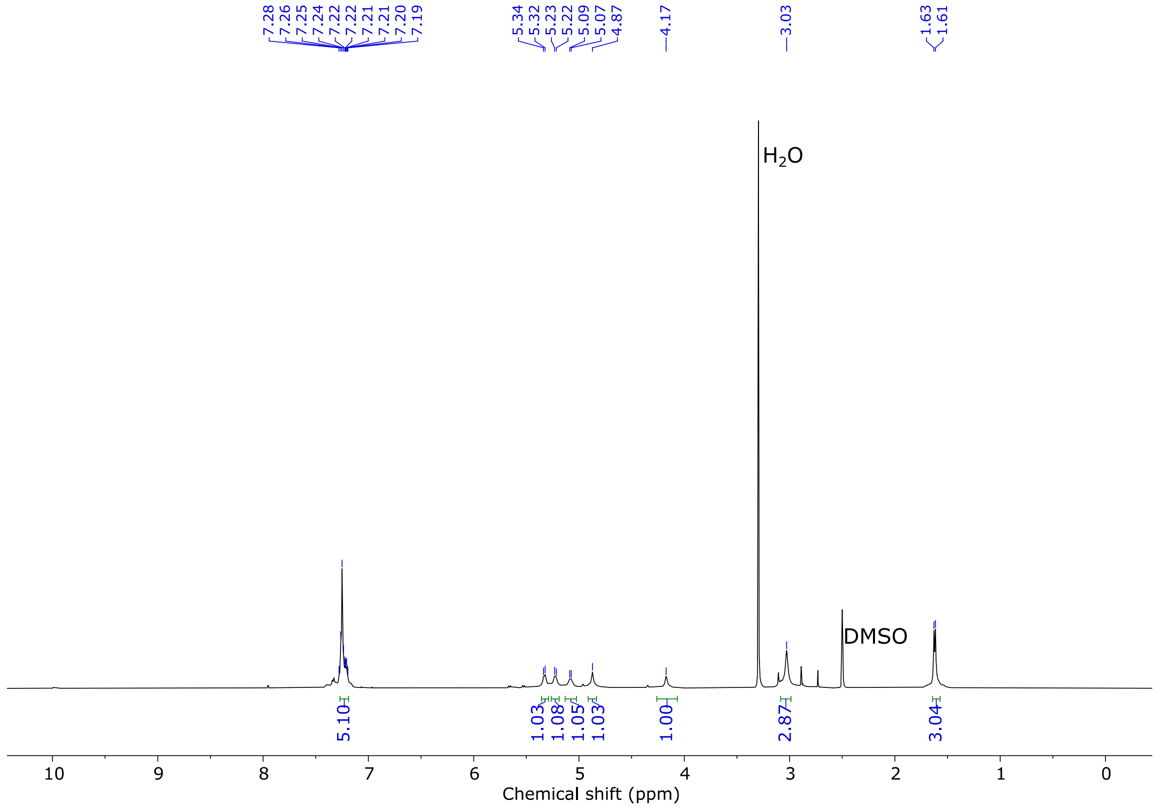

Figure S14: ^{1}H NMR spectrum (500.13 MHz, DMSO-d_6, 30 °C) of **8**.

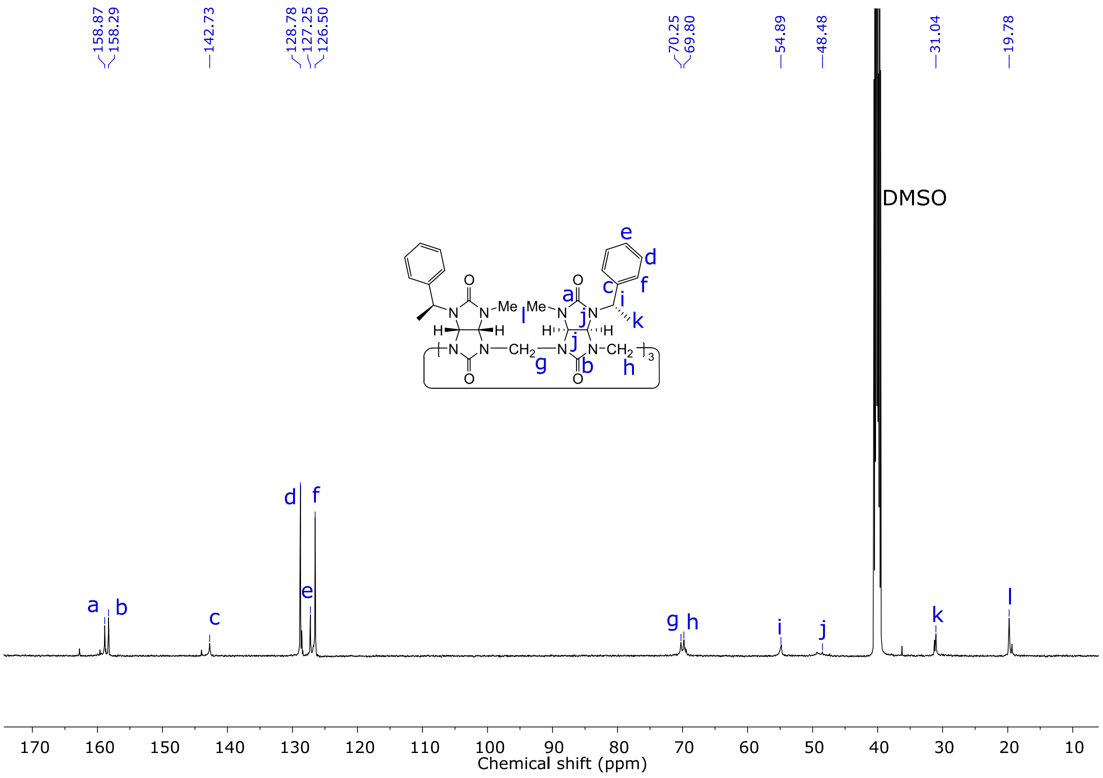

Figure 15: $^{13}C\{^1H\}$ NMR spectrum (125.77 MHz, DMSO-d_6, 30 °C) of **8** (assignment).

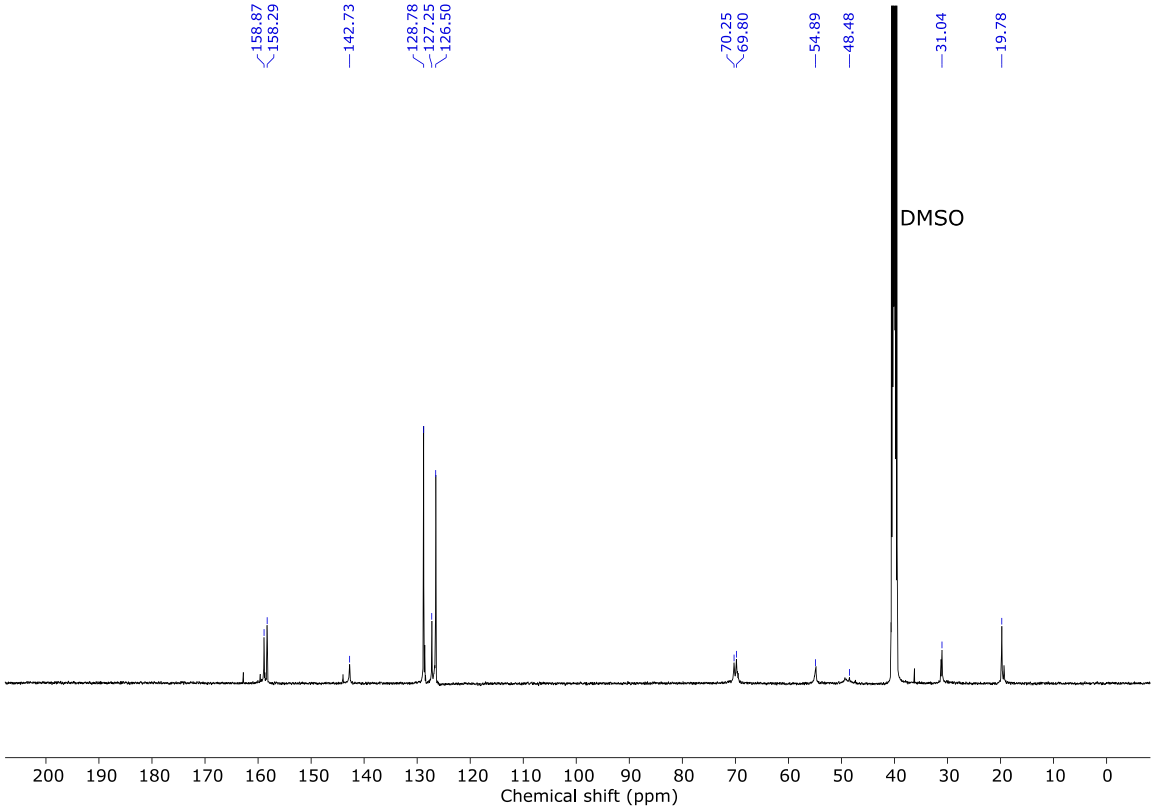

Figure S16: $^{13}C\{^1H\}$ NMR spectrum (125.77 MHz, DMSO-d_6, 30 °C) of **8**.

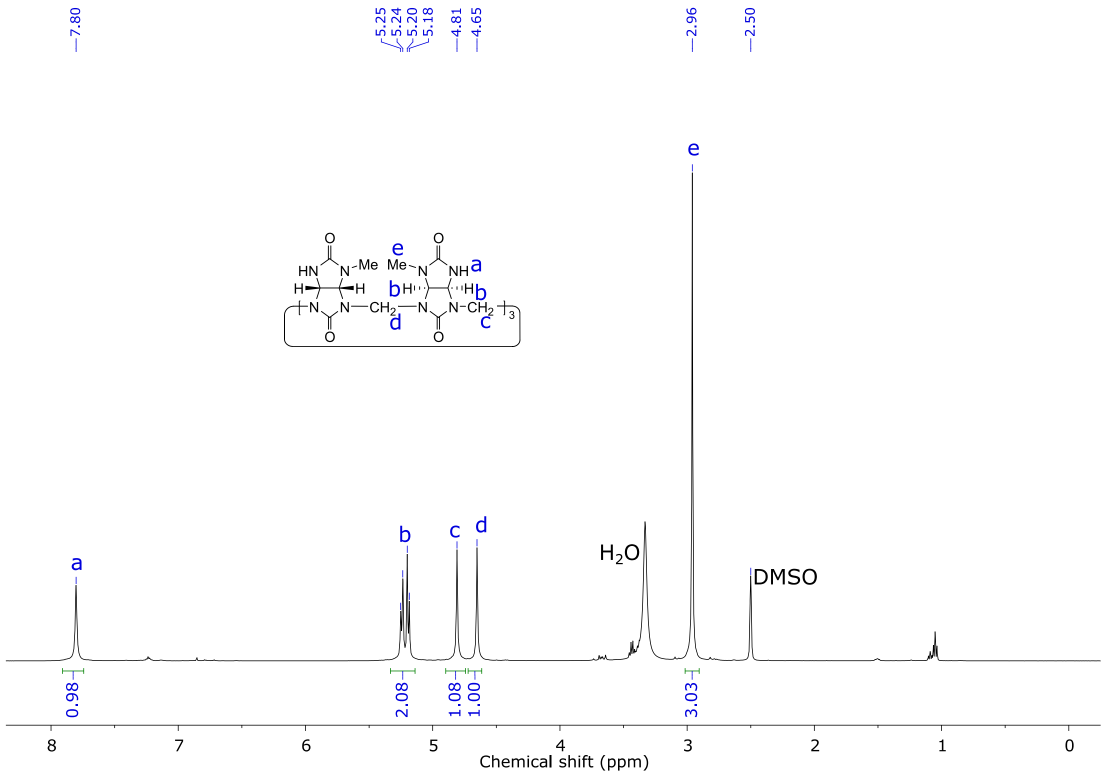

Figure S17: ^{1}H NMR spectrum (500.13 MHz, DMSO-*d*$_6$, 30 °C) of **9** (assignment).

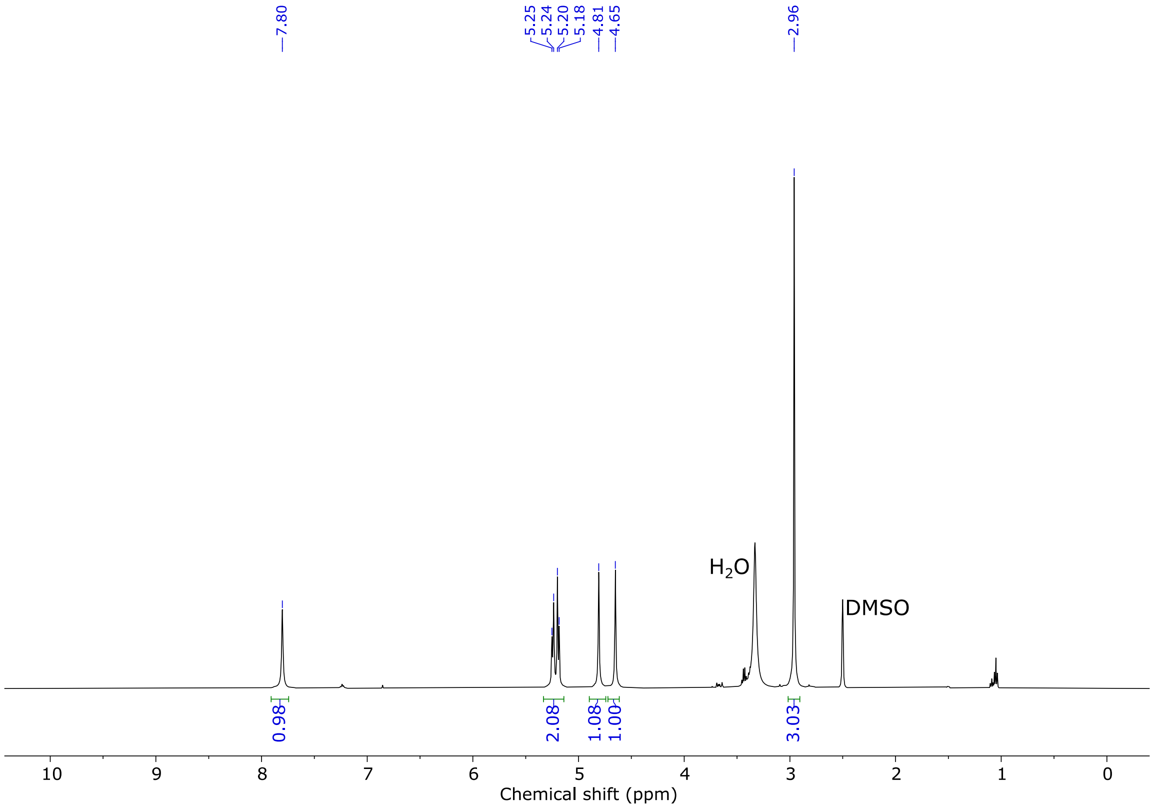

Figure S18: ^{1}H NMR spectrum (500.13 MHz, DMSO-*d*$_6$, 30 °C) of **9**.

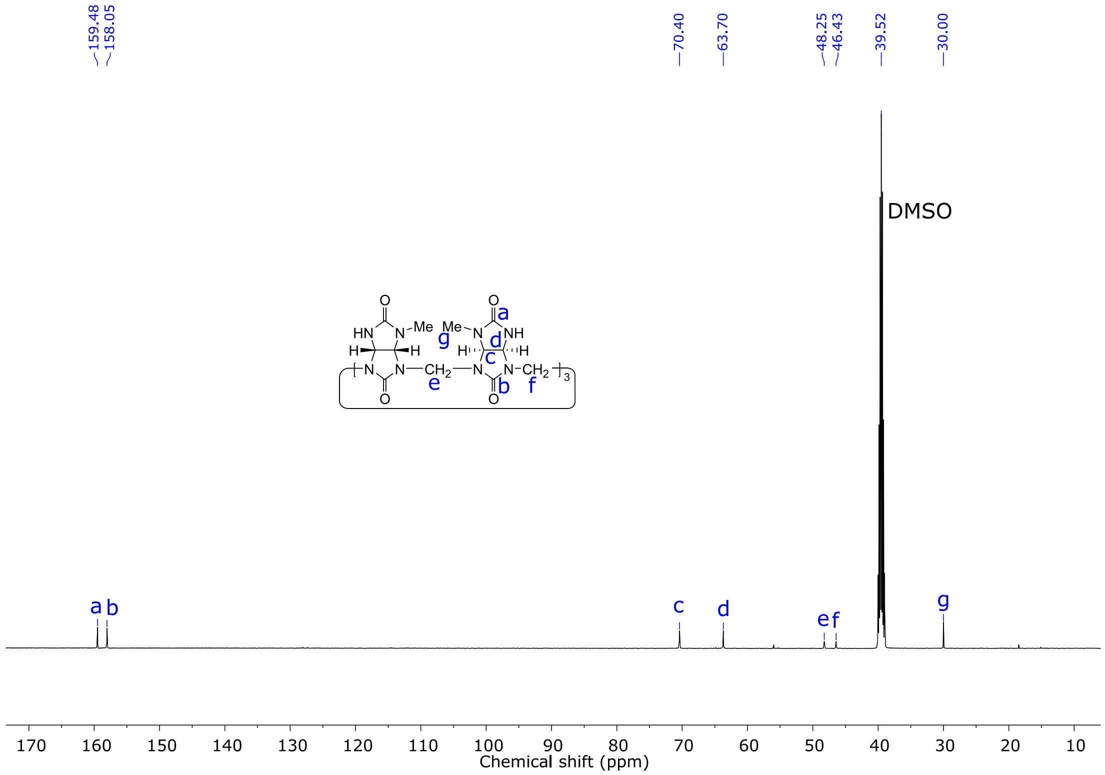

Figure S19: $^{13}C\{^1H\}$ NMR spectrum (125.77 MHz, DMSO-d_6, 30 °C) of **9** (assignment).

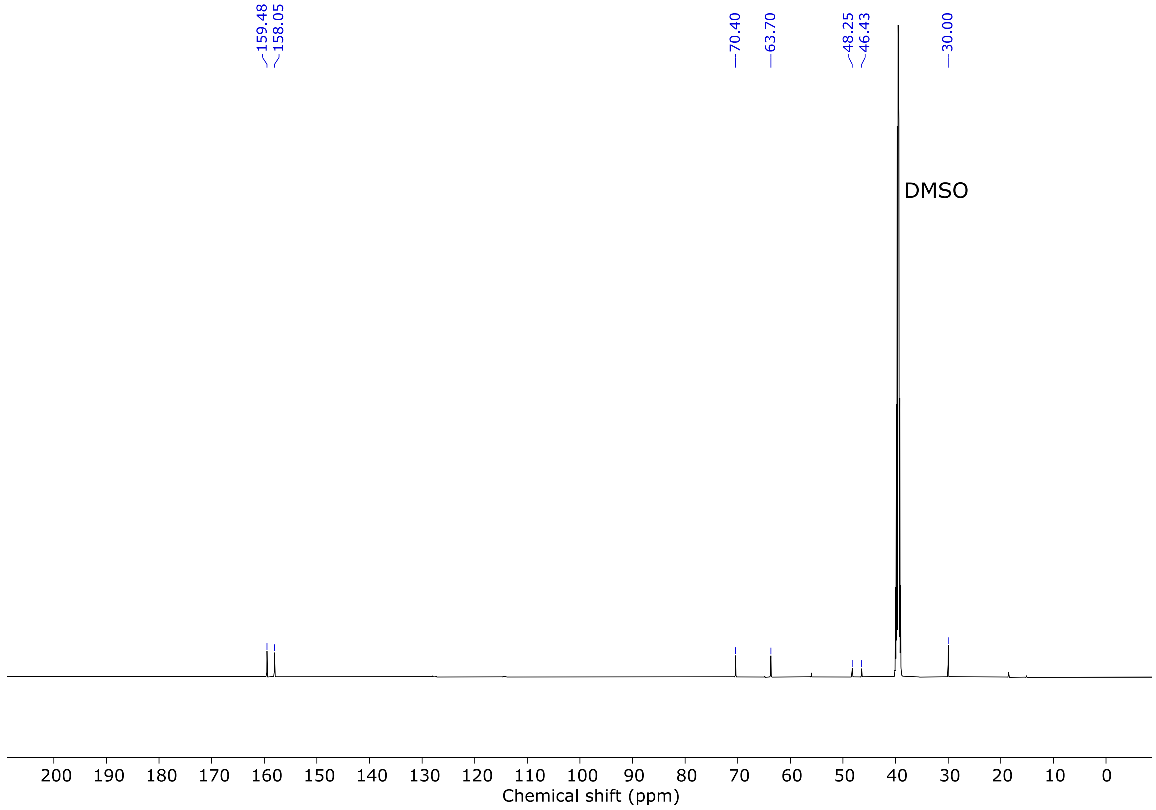

Figure S20: $^{13}C\{^1H\}$ NMR spectrum (125.77 MHz, DMSO-d_6, 30 °C) of **9**.

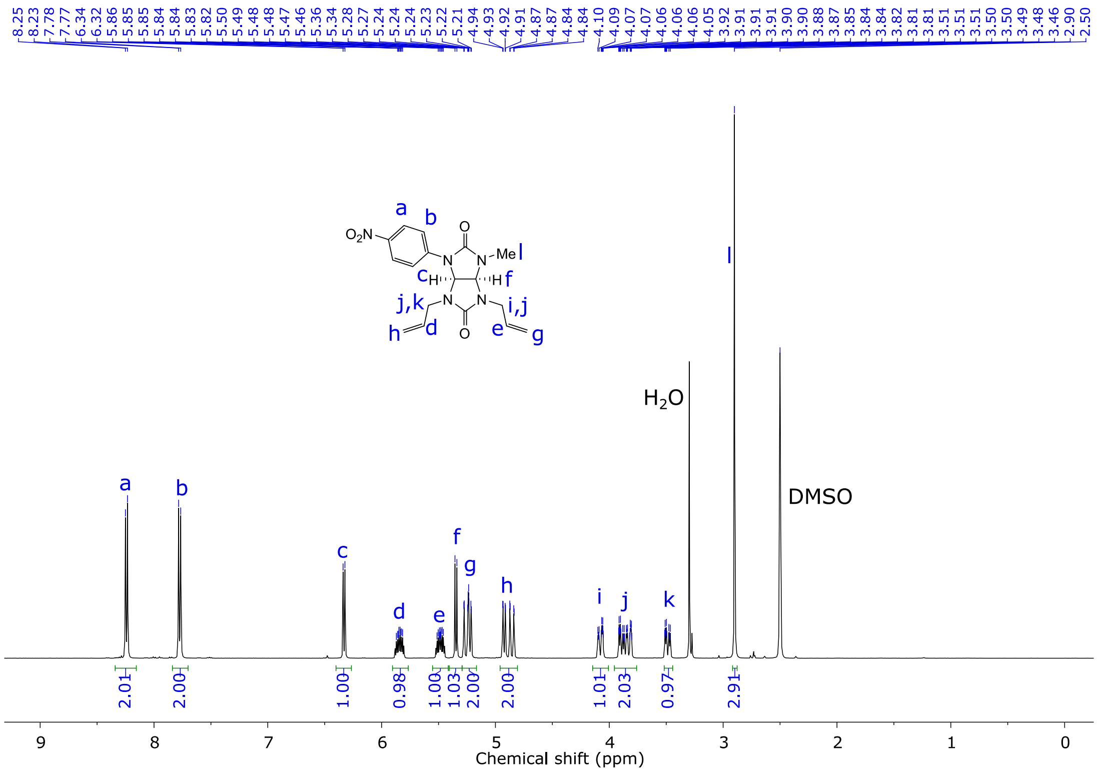

Figure S21:^{1}H NMR spectrum (500.13 MHz, DMSO-*d*$_6$, 30 °C) of **2** (assignment).

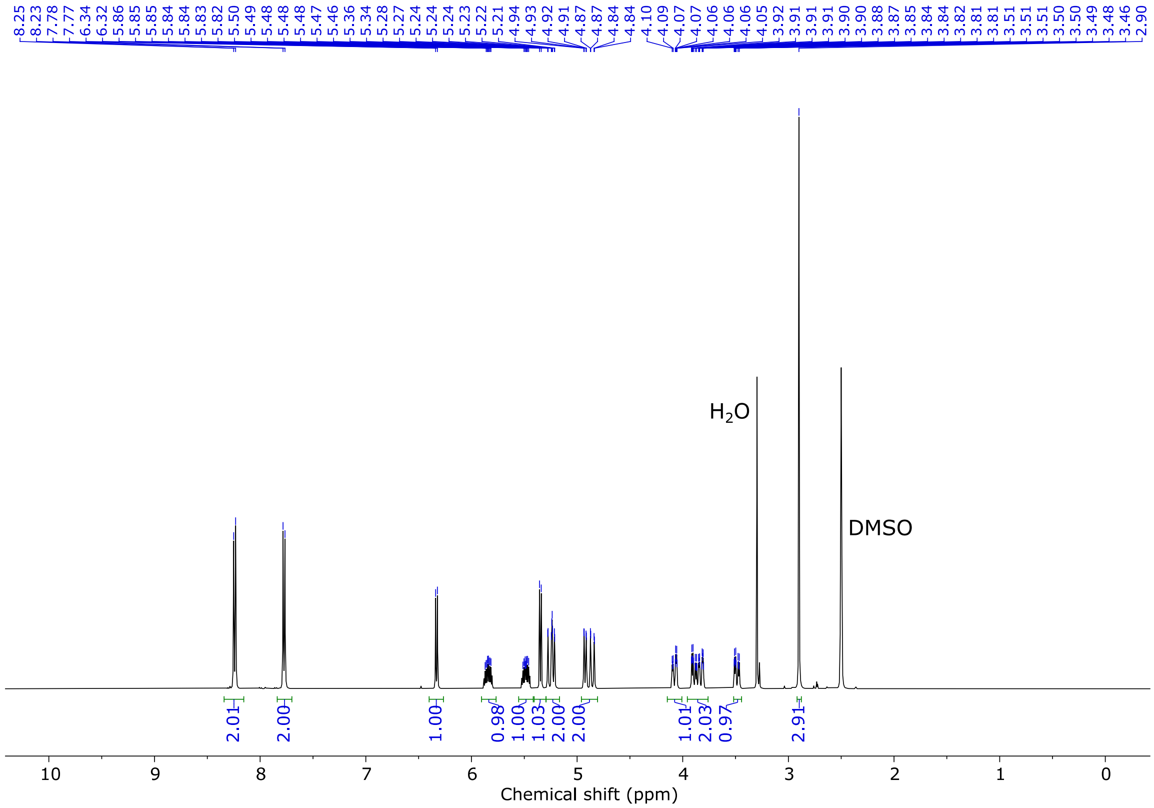

Figure S22:^{1}H NMR spectrum (500.13 MHz, DMSO-*d*$_6$, 30 °C) of **2**.

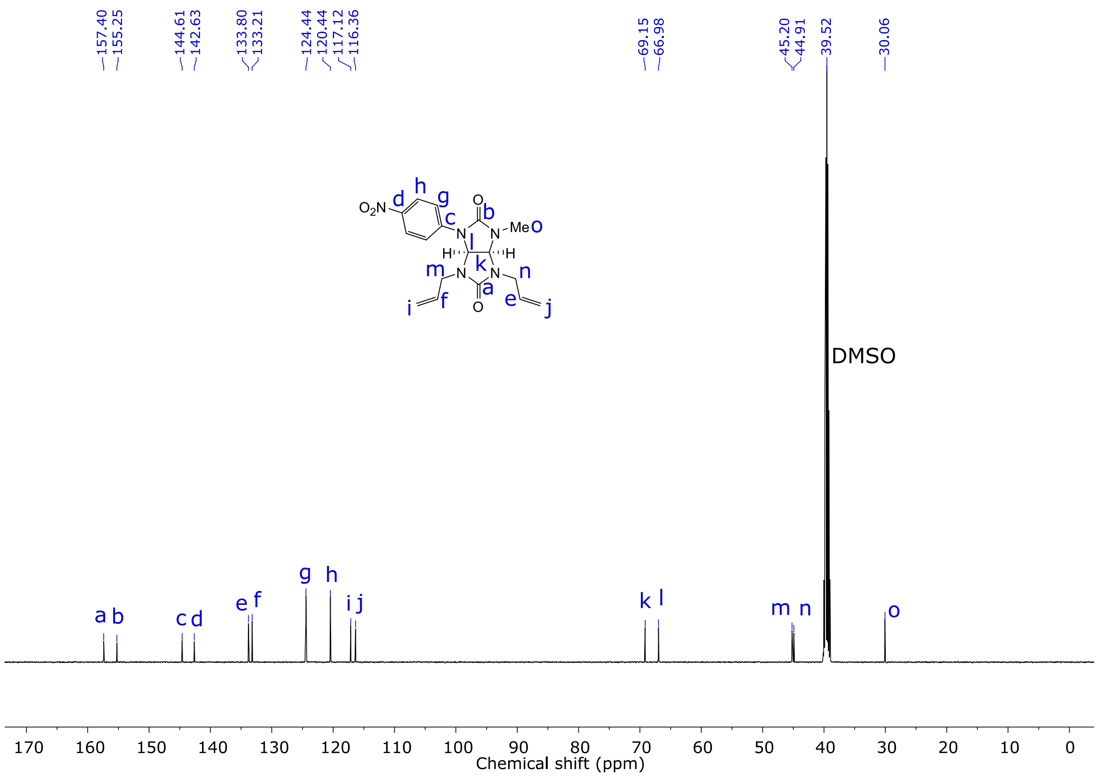

Figure S23: $^{13}C\{^{1}H\}$ NMR spectrum (125.77 MHz, DMSO-d_6, 30 °C) of **2** (assignment).

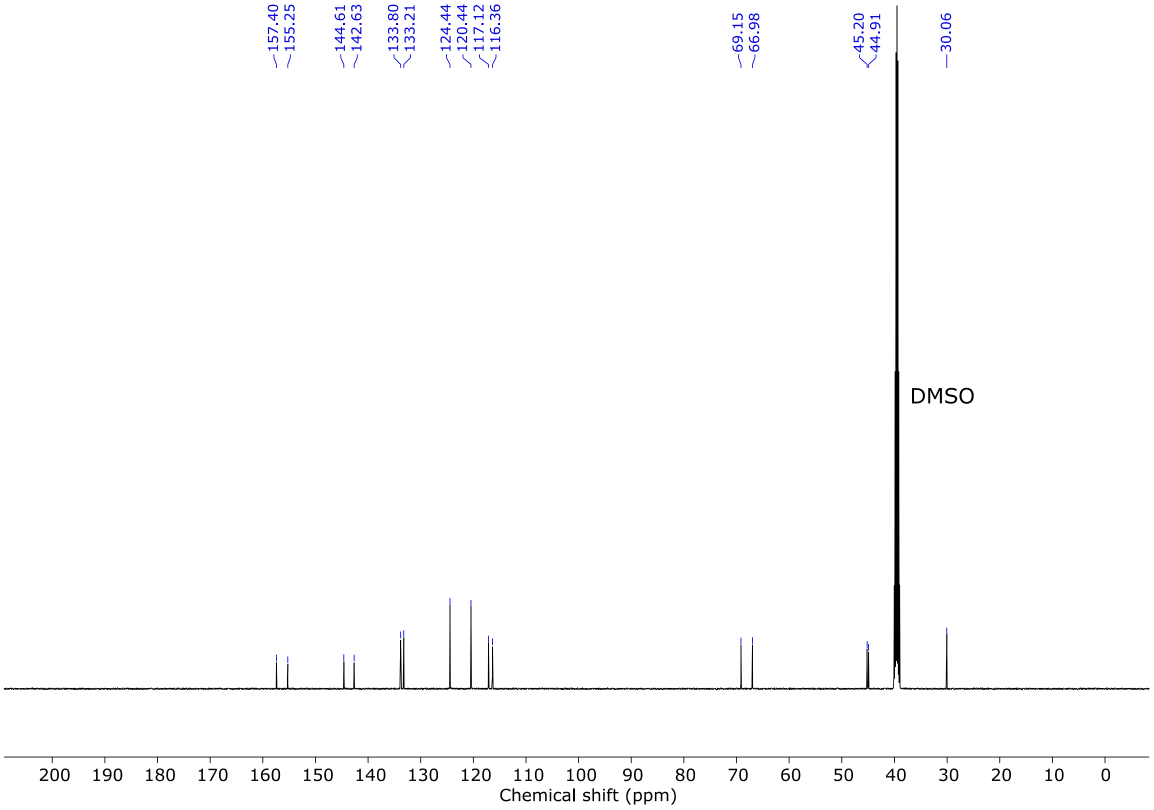

Figure S24: $^{13}C\{^{1}H\}$ NMR spectrum (125.77 MHz, DMSO-d_6, 30 °C) of **2**.

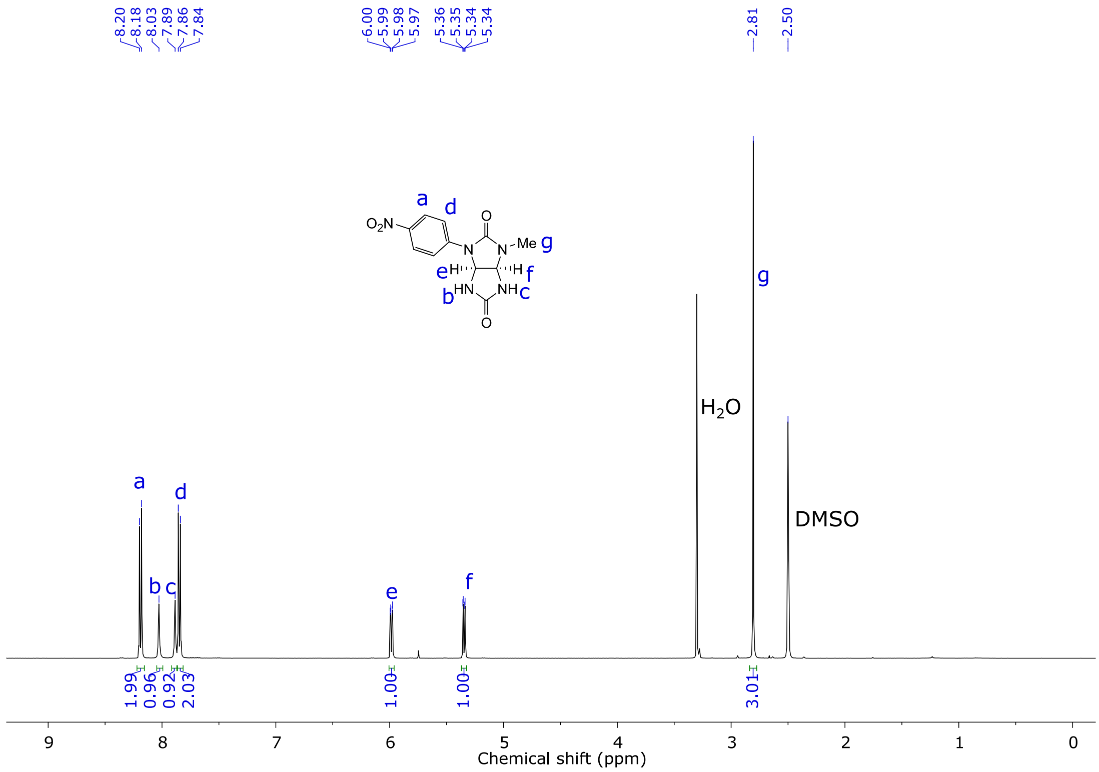

Figure S25: ^{1}H NMR spectrum (500.13 MHz, DMSO-d_6, 30 °C) of **3** (assignment).

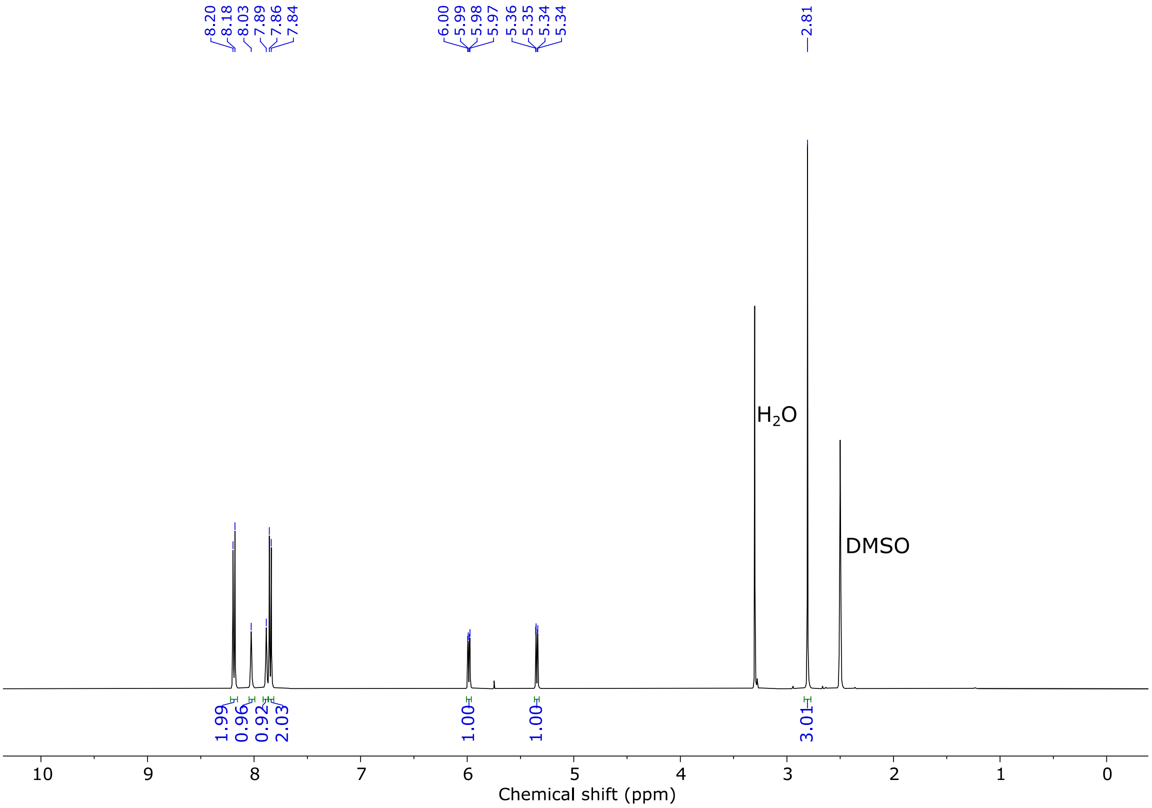

Figure S26: ^{1}H NMR spectrum (500.13 MHz, DMSO-d_6, 30 °C) of **3**.

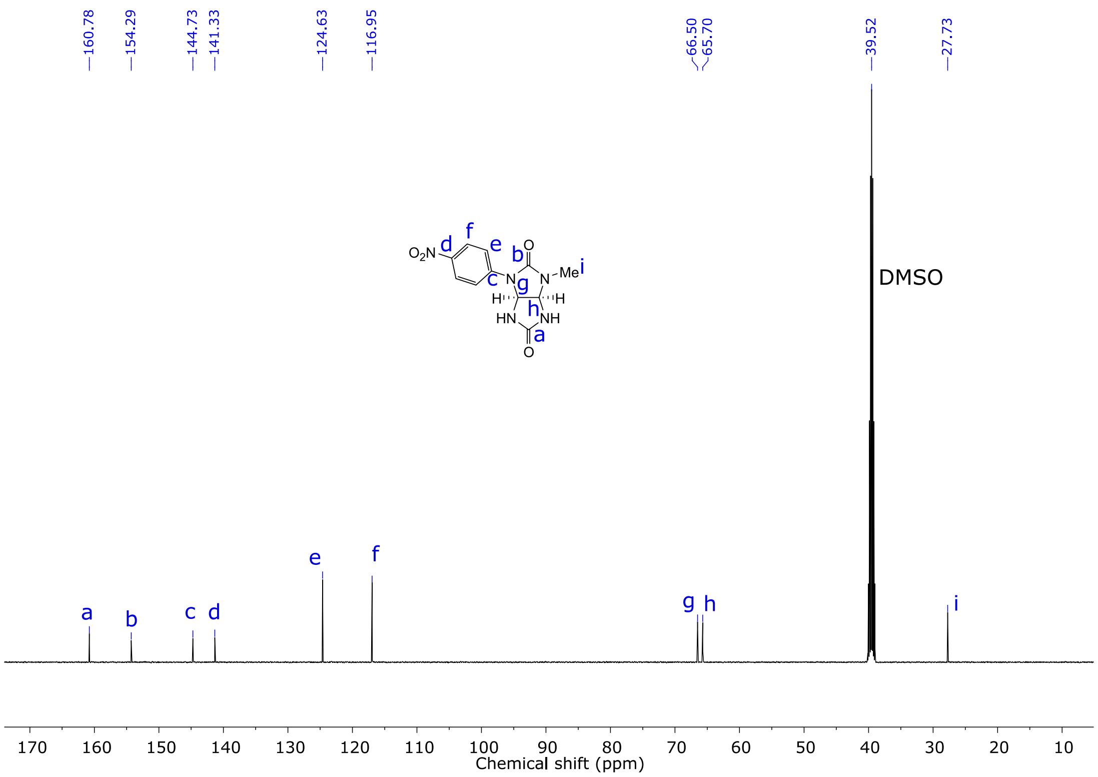

Figure S27: $^{13}C\{^1H\}$ NMR spectrum (125.77 MHz, DMSO-d_6, 30 °C) of **3** (assignment).

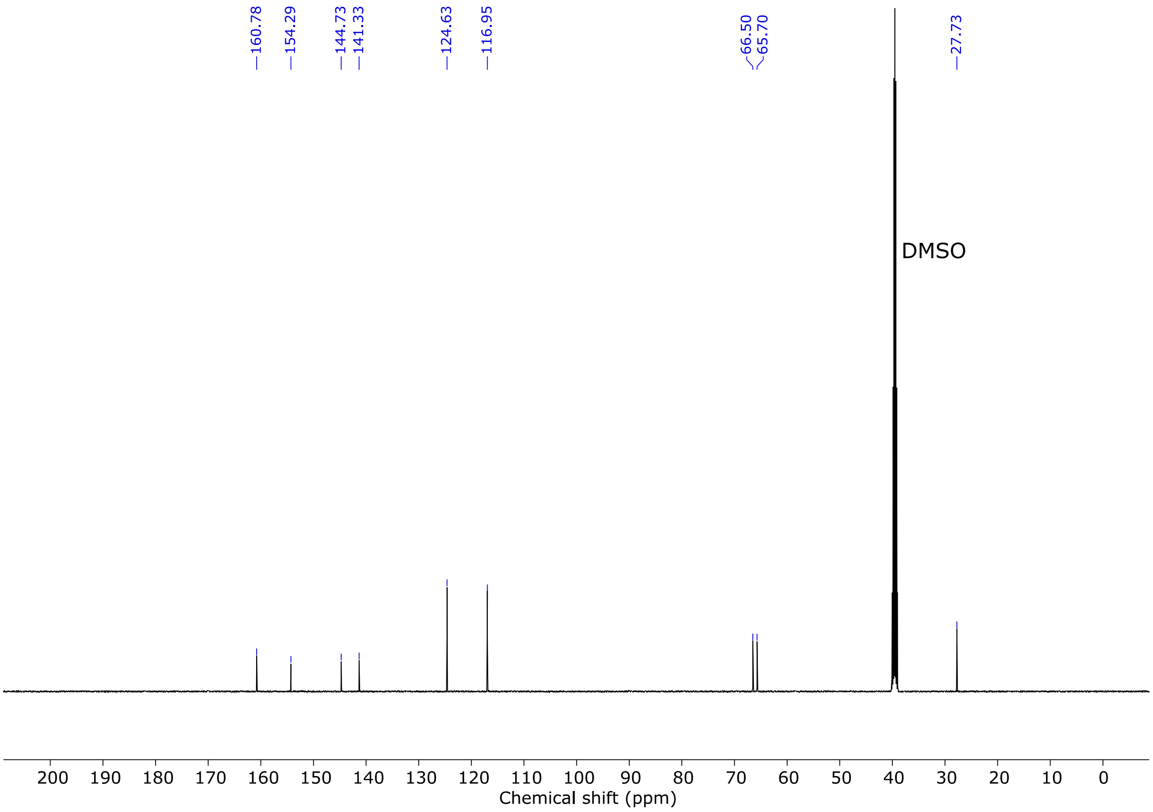

Figure S28: $^{13}C\{^1H\}$ NMR spectrum (125.77 MHz, DMSO-d_6, 30 °C) of **3**.

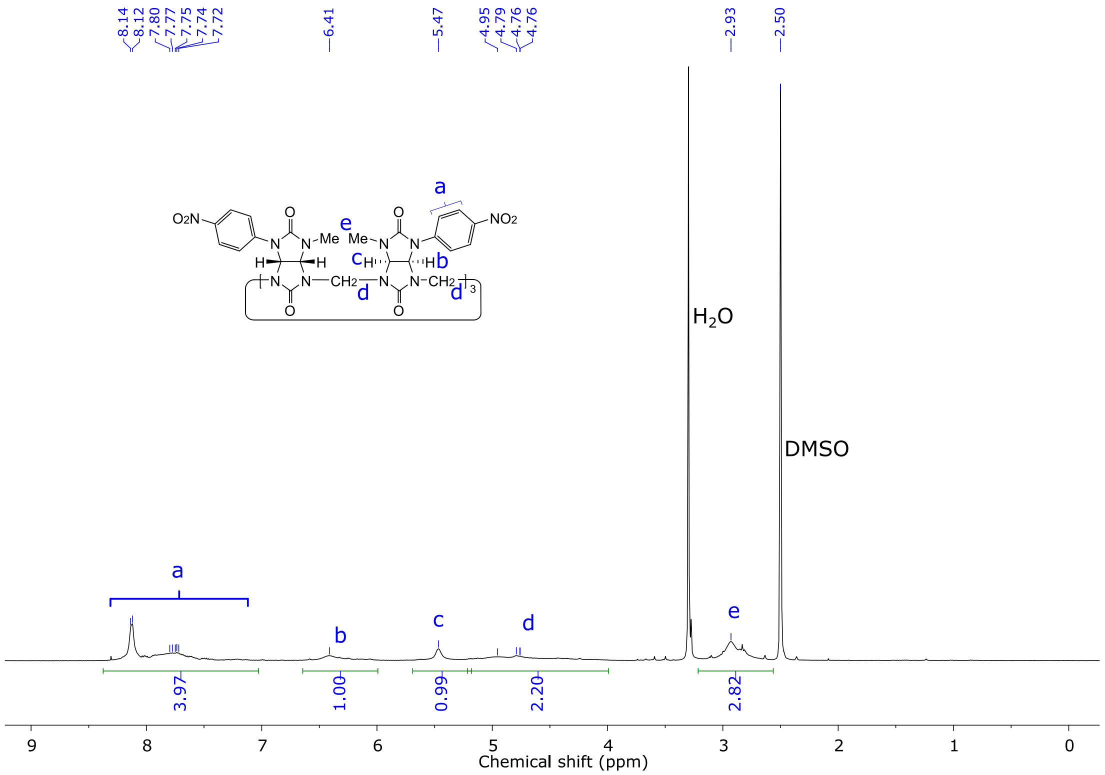

Figure S29: ^{1}H NMR spectrum (500.13 MHz, DMSO-d_6, 30 °C) of **BUI** (assignment).

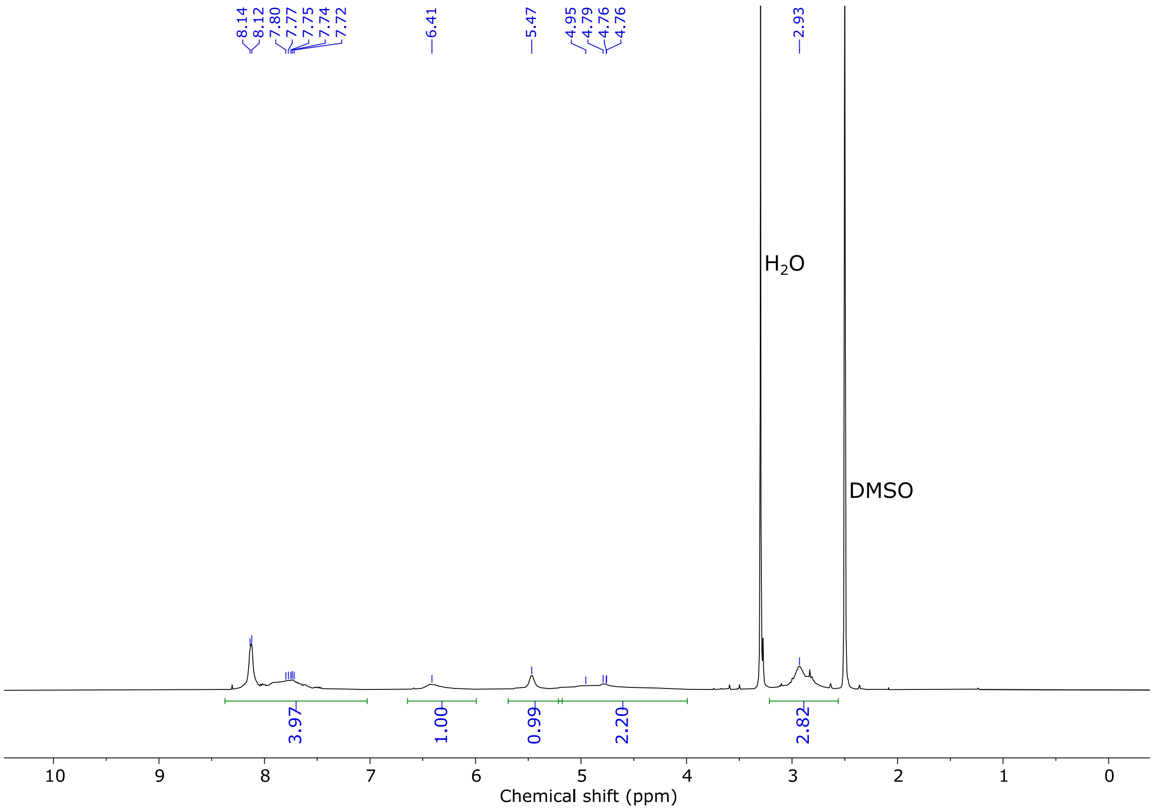

Figure S30: ^{1}H NMR spectrum (500.13 MHz, DMSO-d_6, 30 °C) of **BUI**.

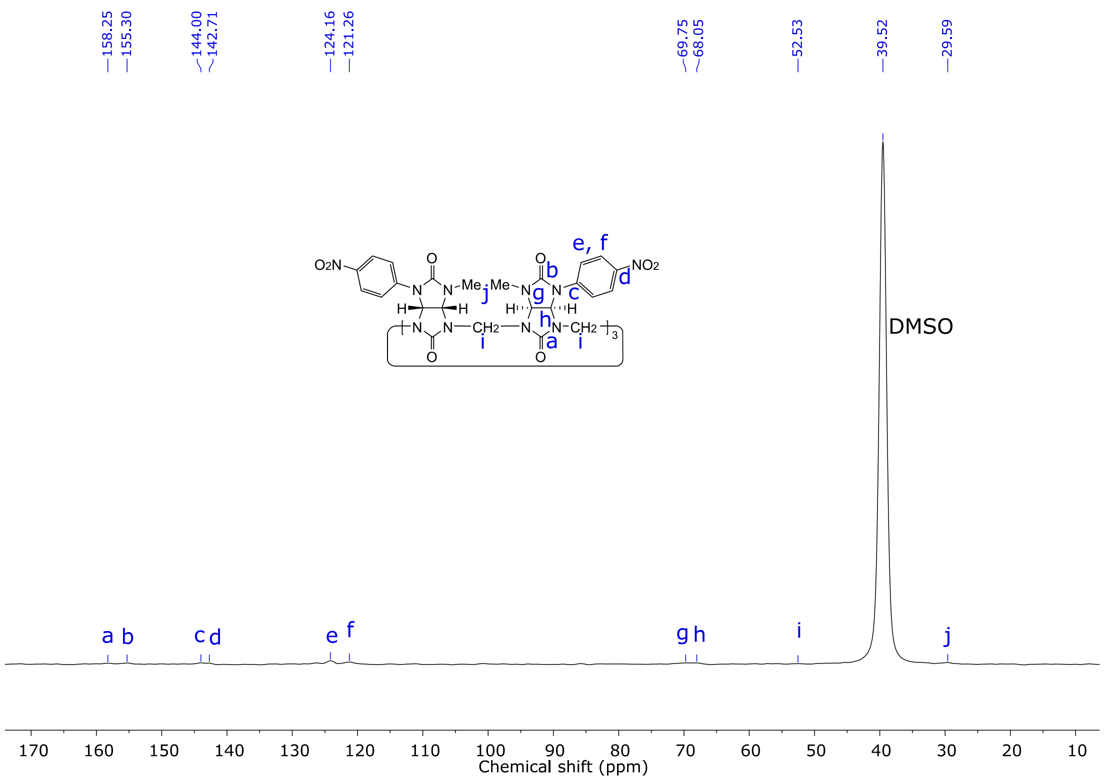

Figure S31: $^{13}C\{^{1}H\}$ NMR spectrum (125.77 MHz, DMSO-d_6, 30 °C) of **BUI** (assignment).

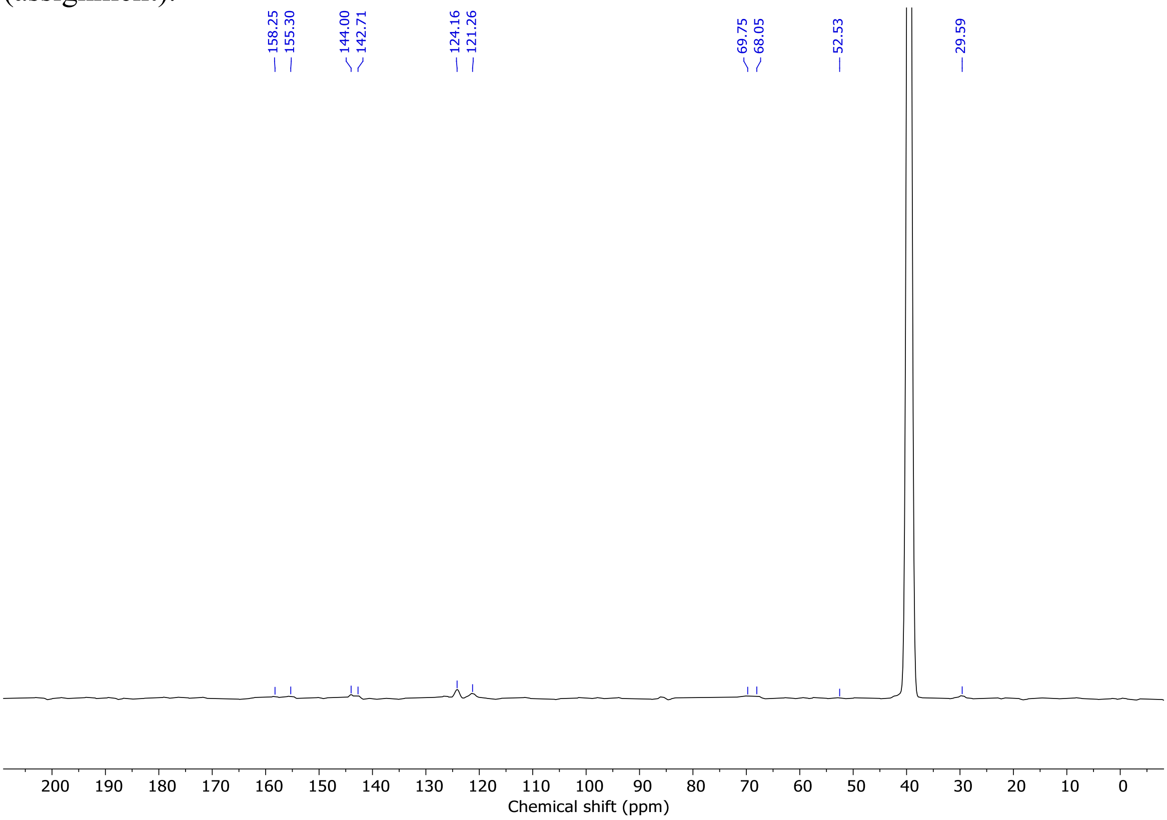

Figure S32: $^{13}C\{^{1}H\}$ NMR spectrum (125.77 MHz, DMSO-d_6, 30 °C) of **BUI**.

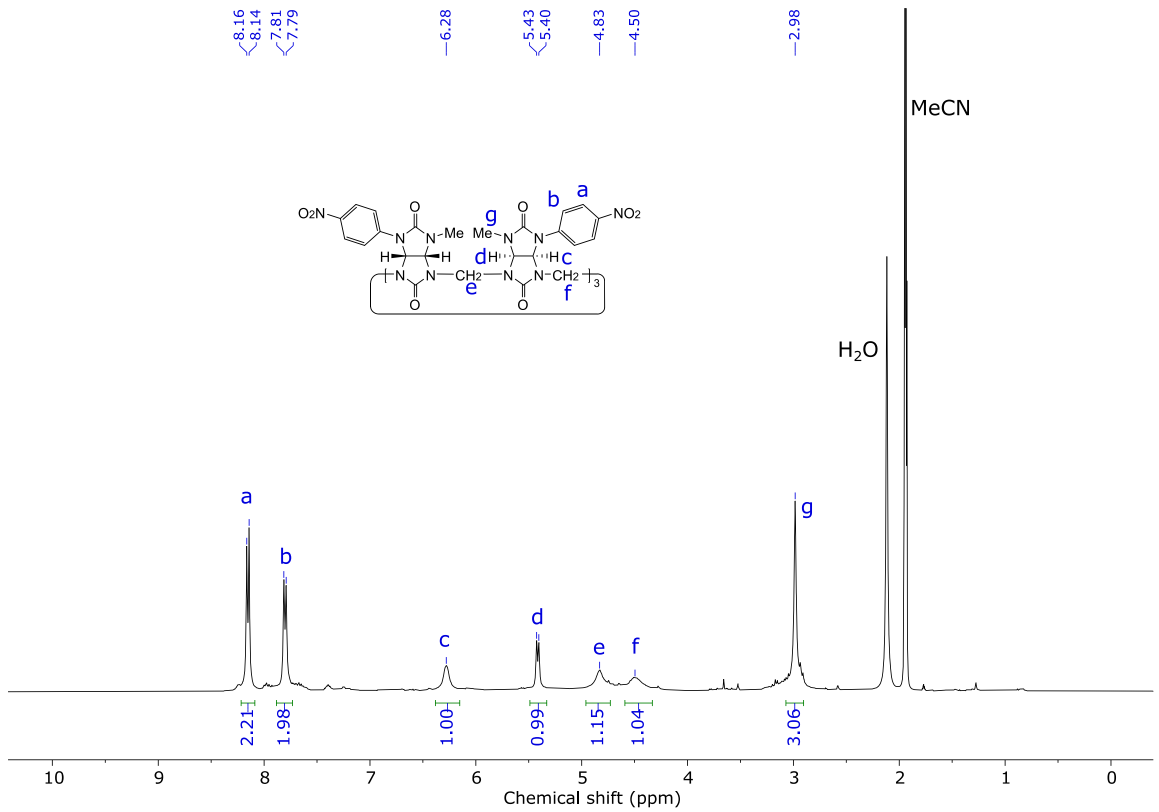

Figure S33: ^{1}H NMR spectrum (400.30 MHz, CD_3CN, 30 °C) of **BUI**.

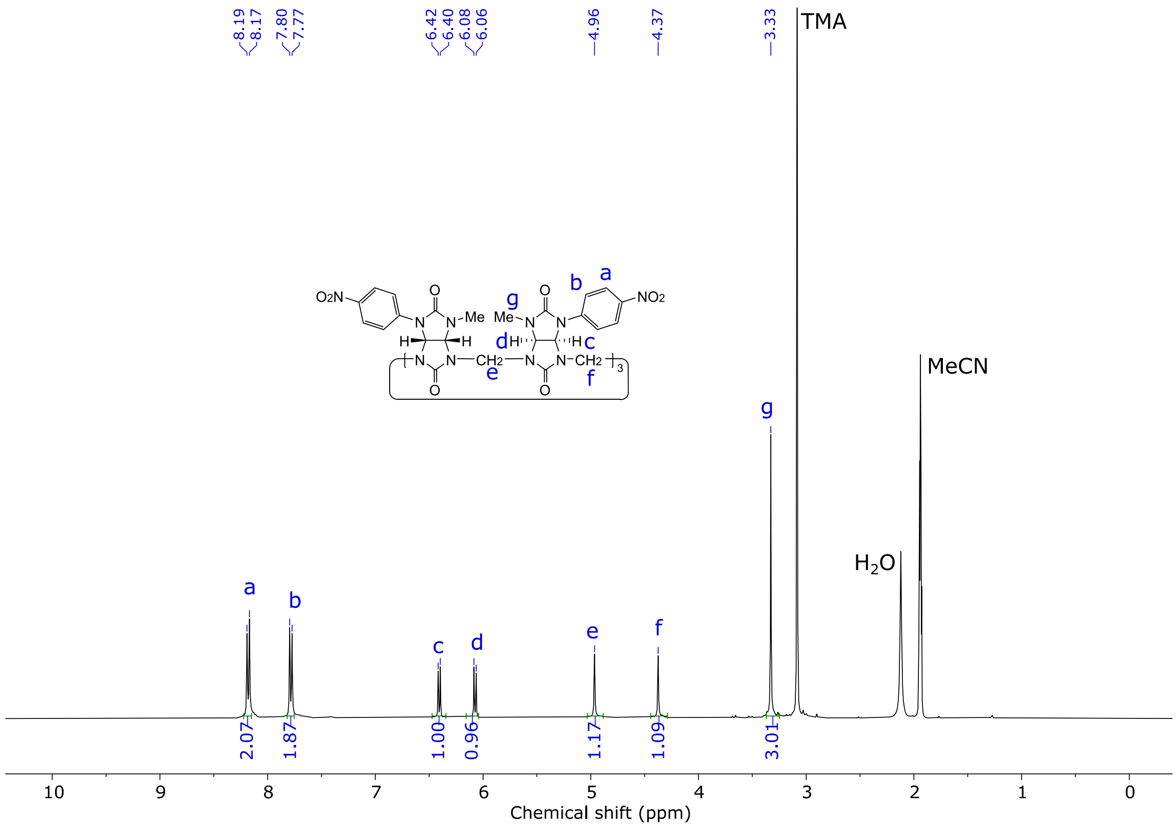

Figure S34: ^{1}H NMR spectrum (400.30 MHz, CD_3CN, 30 °C) of **BUI** in the presence of TMAI.

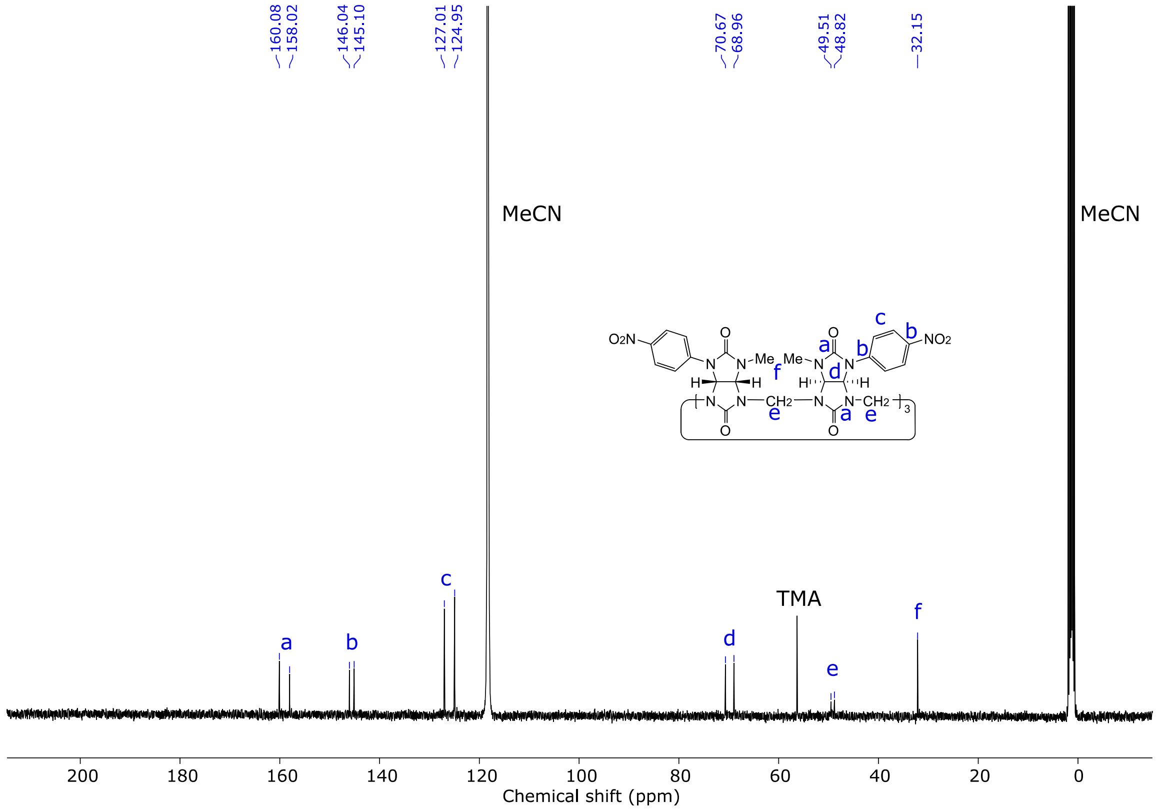

Figure S35: $^{13}C\{^{1}H\}$ NMR spectrum (100.67 MHz, CD_3CN, 30 °C) of **BUI** in the presence of TMAI.

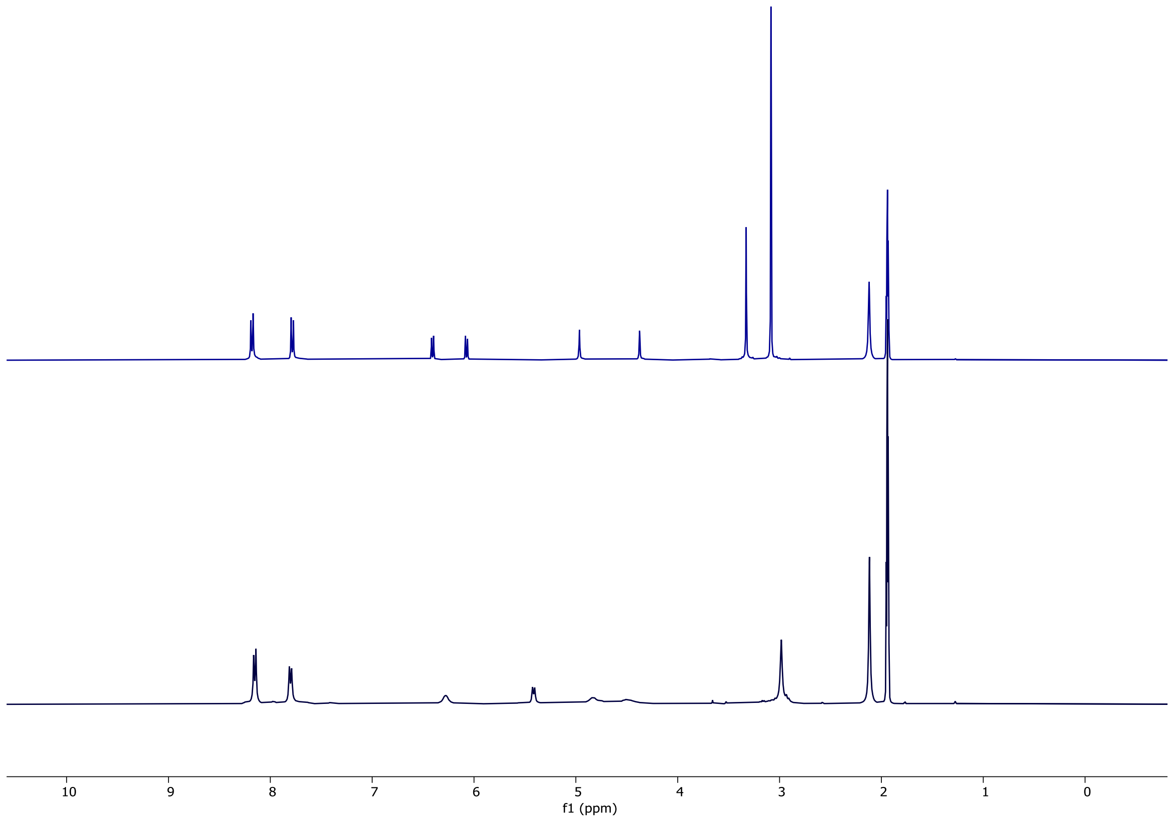

Figure S36: ^{1}H NMR spectrum (400.30 MHz, CD_3CN, 30 °C) of **BUI** without anion and in the presence of TMAI.

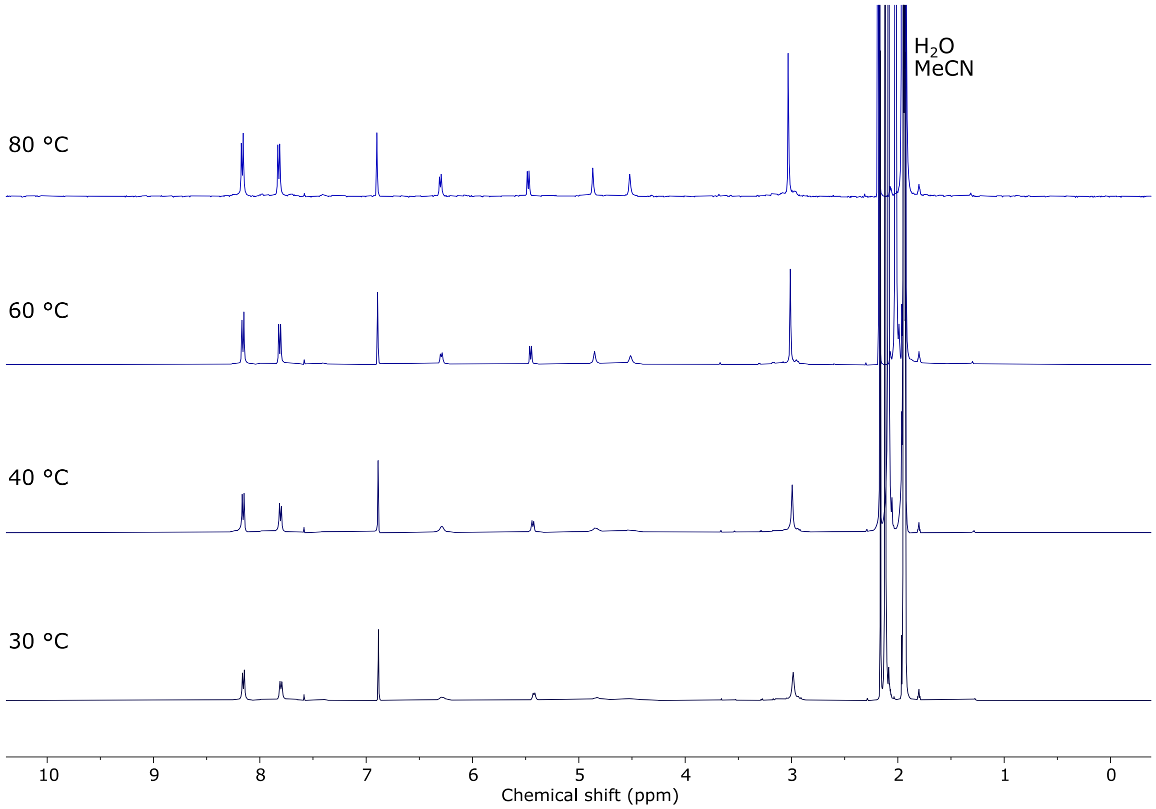

Figure S37: ^{1}H NMR spectrum (400.30 MHz, CD_3CN, 30 °C) of **BUI** at different temperatures.

2. NMR Titrations Experiments

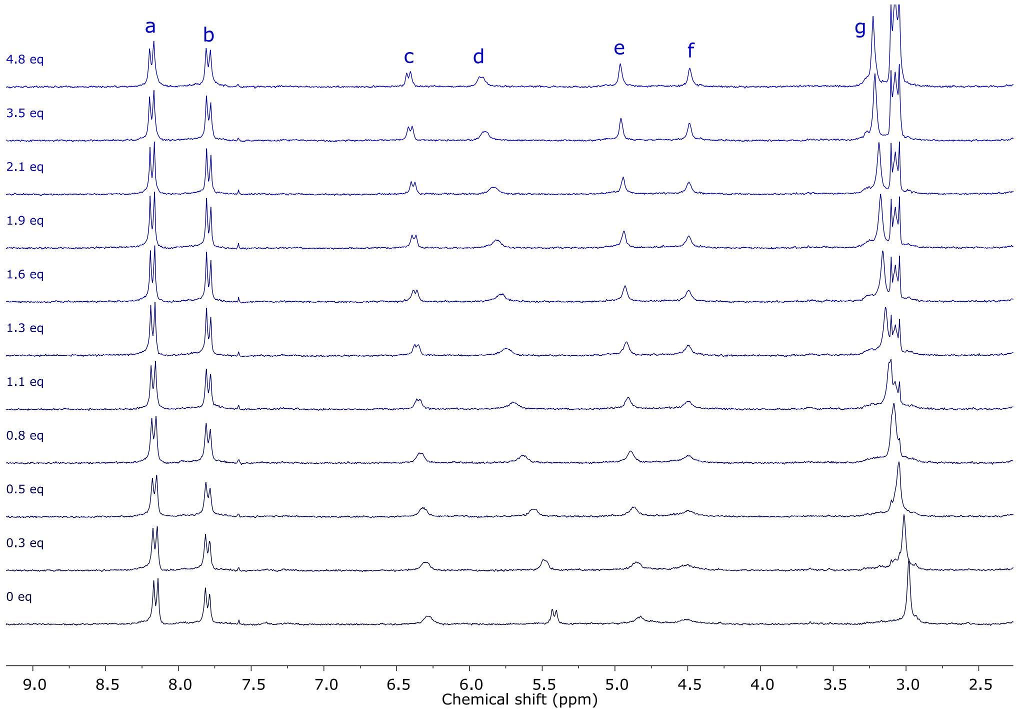

Figure S38: ^{1}H NMR titration (300.13 MHz, CD_3CN, 25 °C) of **BU1** (0.40 mM) with TBACl.

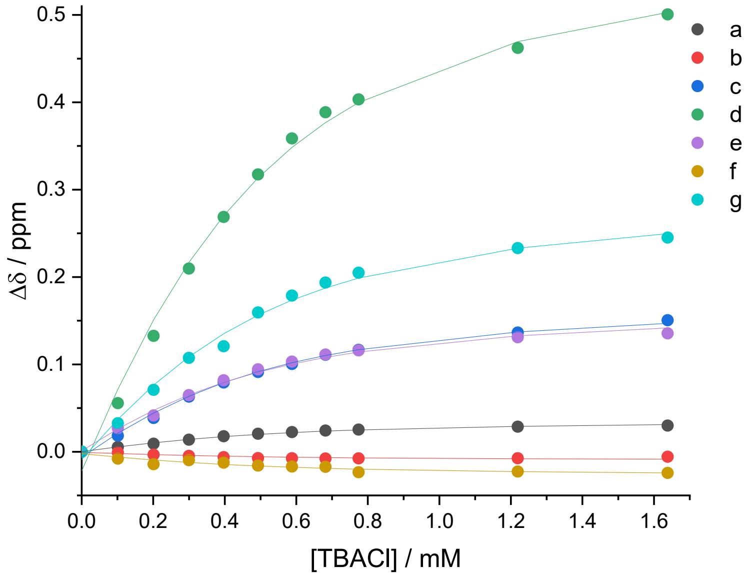

Figure S39: Best fit of the 1:1 binding model to the shift of signals of ^{1}H NMR titration of **BU1** with TBACl.

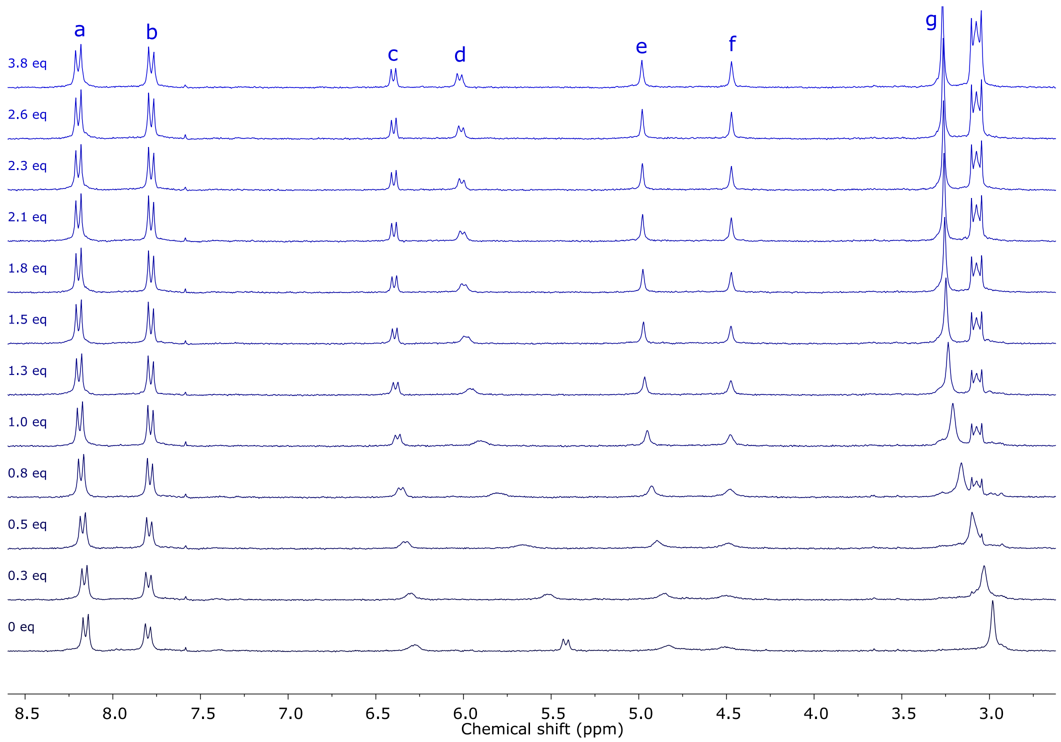

Figure S40: ^{1}H NMR titration (300.13 MHz, CD_3CN, 25 °C) of **BU1** (0.40 mM) with TBABr.

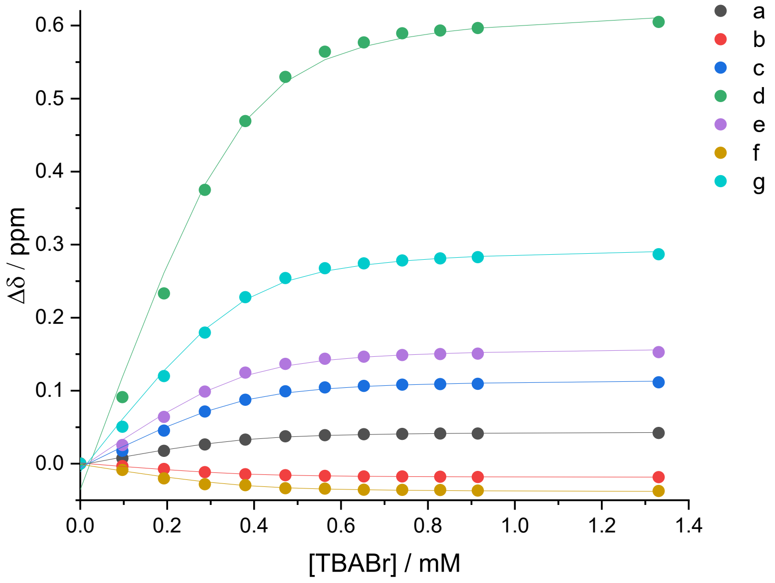

Figure S41: Best fit of the 1:1 binding model to the shift of signals of ^{1}H NMR titration of **BU1** with TBABr.

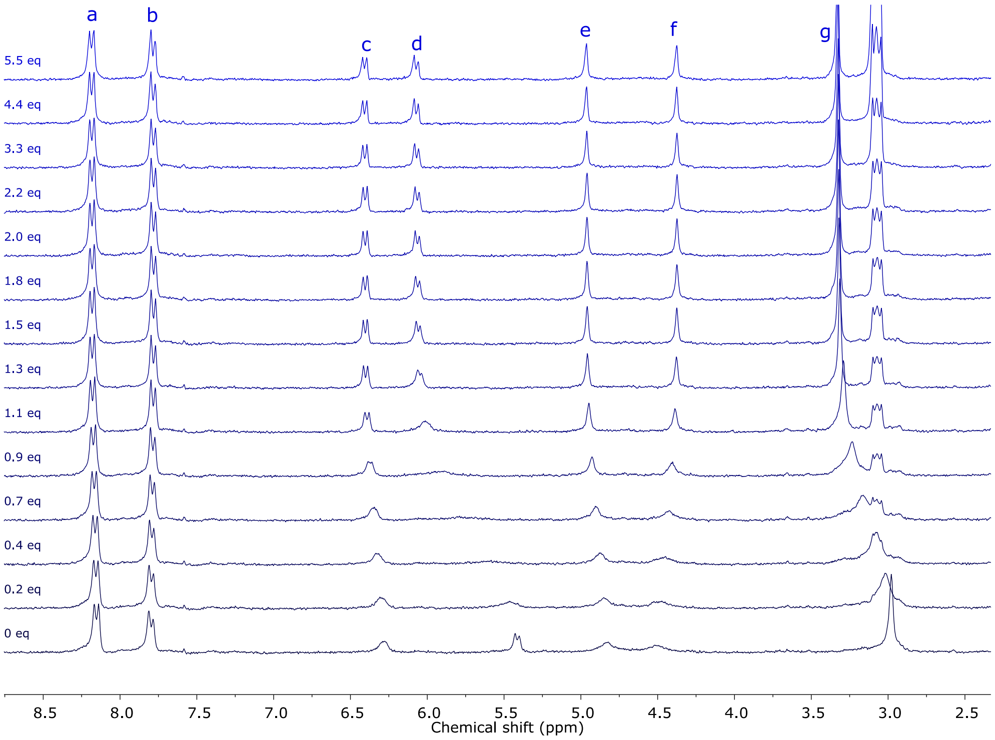

Figure S42: ^{1}H NMR titration (300.13 MHz, CD_3CN, 25 °C) of **BU1** (0.40 mM) with TBAI.

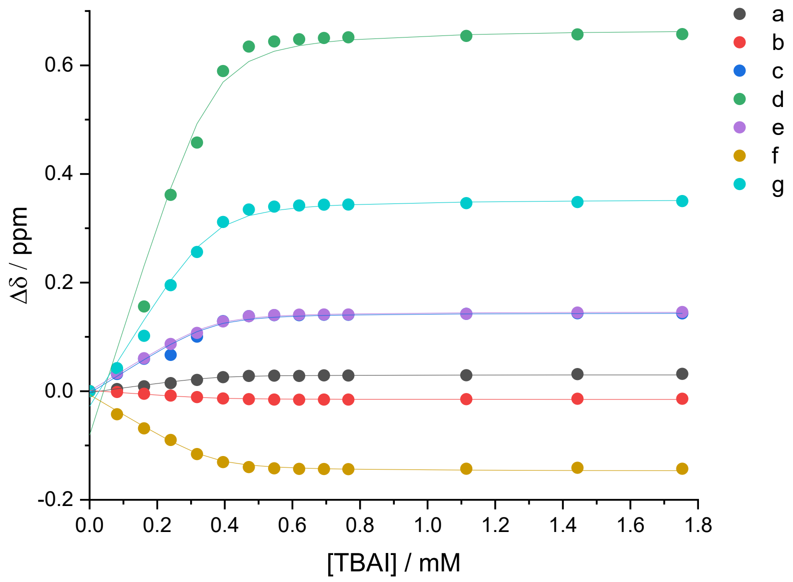

Figure S43: Best fit of the 1:1 binding model to the shift of signals of ^{1}H NMR titration of **BU1** with TBAI.

3. Computational Details

Geometry optimizations of **BU1** were carried out using the semiempirical PM6 method and density functional theory (DFT). The PM6 optimization was performed in Gaussian 16 (rev. C.01) with a CPCM(acetonitrile) solvation model. The resulting geometry was then used as the starting structure for DFT optimization at the BLYP/def2-SVP level with D3(0) dispersion correction and the same CPCM(acetonitrile) solvation model in ORCA (6.1.0). Frequency calculations verified that the DFT-optimized structure corresponds to a true minimum.

1. **the number of imaginary frequencies**

BLYP – zero negative frequencies

2. **computed total energies of target or optimized structures:**

A. DFT (BLYP)
Total electronic energy (BLYP-D3(0)/def2-SVP, CPCM(MeCN)):
E = −6225.317874 a.u.
B. Semiempirical (PM6)
Total electronic energy (PM6, CPCM(MeCN)):
E = −0.771118 a.u.

PM6-optimized structure of **BU1** (CPCM acetonitrile):

C	2.91824	2.79806	1.92221
H	1.85607	2.97448	2.23881
C	3.34012	1.25669	1.85339
H	2.49409	0.53458	1.99134
N	3.90275	1.12296	0.47462
N	3.19361	3.31054	0.55365
N	3.87853	3.37075	2.92245
N	4.32437	1.12220	2.94524
C	4.76943	2.35991	3.44141
C	3.73796	2.29874	-0.29047
O	5.66638	2.54726	4.23768
O	4.02186	2.43222	-1.46631
C	5.02816	-0.14246	3.22953
H	5.81475	-0.36049	2.47881
H	5.50118	-0.09739	4.22798
C	0.67223	-3.92775	1.77441
H	1.18453	-3.01643	2.17698
C	-0.91110	-3.77379	1.60866
H	-1.30000	-2.73913	1.79720
N	-1.14932	-4.19859	0.19596
N	1.13619	-4.23140	0.39653
N	0.80895	-5.09226	2.70724
N	-1.45992	-4.71049	2.61609
C	-0.48455	-5.61376	3.09254
C	0.06122	-4.34432	-0.52151
O	-0.66927	-6.58705	3.79152
O	0.16735	-4.53240	-1.72174
C	-2.89216	-5.07046	2.63284
H	-3.15651	-5.83013	1.87653
H	-3.15621	-5.47518	3.62879
C	-3.81982	0.85769	1.99290
H	-3.43302	-0.18529	2.13532
C	-2.69748	1.99006	2.02857
H	-1.65654	1.58878	2.13689
N	-2.82189	2.66688	0.69992
N	-4.46132	1.03698	0.66229

N	-4.72394	1.24129	3.12147
N	-3.09581	2.89963	3.13490
C	-4.28316	2.44616	3.77697
C	-3.86010	2.09123	-0.07887
O	-4.84769	2.99336	4.69788
O	-4.14940	2.41027	-1.21807
C	-2.02314	3.49993	3.96372
H	-2.45648	4.27418	4.62372
H	-1.49572	2.76209	4.59267
C	0.67047	3.92919	-1.77450
H	1.18333	3.01826	-2.17727
C	-0.91275	3.77427	-1.60855
H	-1.30106	2.73942	-1.79726
N	-1.15098	4.19861	-0.19572
N	1.13441	4.23312	-0.39669
N	0.80636	5.09390	-2.70719
N	-1.46224	4.71094	-2.61562
C	-0.48750	5.61486	-3.09203
C	0.05957	4.34504	0.52159
O	-0.67285	6.58830	-3.79064
O	0.16576	4.53299	1.72184
C	-2.89477	5.06977	-2.63256
H	-3.15932	5.83091	-1.87782
H	-3.15944	5.47213	-3.62930
C	-3.82014	-0.85892	-1.99219
H	-3.43388	0.18426	-2.13461
C	-2.69724	-1.99070	-2.02803
H	-1.65649	-1.58895	-2.13648
N	-2.82116	-2.66762	-0.69939
N	-4.46154	-1.03863	-0.66160
N	-4.72421	-1.24293	-3.12067
N	-3.09524	-2.90046	-3.13434
C	-4.28294	-2.44761	-3.77620
C	-3.85953	-2.09250	0.07957
O	-4.84738	-2.99517	-4.69696
O	-4.14847	-2.41167	1.21880
C	-2.02238	-3.50010	-3.96338
H	-2.45536	-4.27475	-4.62314
H	-1.49567	-2.76198	-4.59260
C	2.91998	-2.79851	-1.92316
H	1.85774	-2.97502	-2.23945
C	3.34199	-1.25713	-1.85494
H	2.49581	-0.53499	-1.99162
N	3.90725	-1.12399	-0.47726
N	3.19571	-3.31064	-0.55455
N	3.88015	-3.37154	-2.92336
N	4.32403	-1.12245	-2.94890
C	4.77039	-2.36071	-3.44333
C	3.74143	-2.29892	0.28878
O	5.66717	-2.54828	-4.23970
O	4.02638	-2.43208	1.46440
C	5.03155	0.14091	-3.22988
H	5.82045	0.35412	-2.48036
H	5.50223	0.09770	-4.22954
C	2.53422	4.50646	-0.00760
H	3.12198	4.84434	-0.90228
H	2.54973	5.32214	0.76517

C	-2.44999	4.08918	0.49031
H	-3.23183	4.59127	-0.14058
H	-2.37667	4.63584	1.46980
C	-5.27889	-0.00086	0.00054
H	-5.93458	0.49786	-0.76357
H	-5.93418	-0.49969	0.76493
C	-2.44848	-4.08973	-0.48985
H	-2.37511	-4.63635	-1.46937
H	-3.22998	-4.59220	0.14119
C	2.53584	-4.50589	0.00749
H	2.55082	-5.32202	-0.76481
H	3.12352	-4.84355	0.90230
C	4.73503	0.00075	-0.00063
H	5.39401	0.37160	-0.83860
H	5.39375	-0.36846	0.83840
H	4.30631	-0.98451	3.22279
H	-1.27689	3.99266	3.30534
H	-3.50551	-4.16129	2.46117
H	4.31217	0.98510	-3.21903
H	-1.27556	-3.99215	-3.30514
H	-3.50745	4.16057	-2.45874
C	-5.90632	0.52839	3.46319
C	-6.97830	1.19879	4.09592
C	-5.99181	-0.85586	3.19675
C	-8.12200	0.49707	4.44584
H	-6.91195	2.27060	4.31485
C	-7.13635	-1.55770	3.54833
H	-5.15661	-1.39780	2.73370
C	-8.20707	-0.88364	4.16977
H	-8.96264	1.00799	4.93225
H	-7.21235	-2.63447	3.34996
C	2.02945	-5.47344	3.32551
C	2.15497	-6.76403	3.89135
C	3.11652	-4.57232	3.37179
C	3.34912	-7.14853	4.48118
H	1.31228	-7.46520	3.86679
C	4.30992	-4.95622	3.96632
H	3.03789	-3.56010	2.95497
C	4.43327	-6.24641	4.52063
H	3.45863	-8.15003	4.91631
H	5.15726	-4.26049	4.01051
C	3.87889	4.72580	3.34168
C	5.05697	5.29846	3.87754
C	2.70103	5.49900	3.23731
C	5.05989	6.62364	4.28283
H	5.96994	4.69980	3.97669
C	2.70389	6.82439	3.64554
H	1.76731	5.05961	2.86100
C	3.88433	7.39563	4.16421
H	5.97015	7.07885	4.69280
H	1.79250	7.43088	3.57247
N	-9.40962	-1.61869	4.53501
O	-9.46478	-2.81927	4.29041
O	-10.32920	-1.01154	5.07453
N	5.68607	-6.64889	5.14120
O	6.58971	-5.82338	5.23345
O	5.79944	-7.79877	5.55315

N	3.88863	8.78665	4.58627
O	2.84637	9.43083	4.51081
O	4.93399	9.27573	5.00393
C	2.02663	5.47623	-3.32514
C	3.11426	4.57582	-3.37190
C	2.15136	6.76721	-3.89031
C	4.30742	4.96077	-3.96621
H	3.03621	3.56329	-2.95568
C	3.34527	7.15276	-4.47990
H	1.30822	7.46781	-3.86538
C	4.43000	6.25134	-4.51981
H	5.15519	4.26558	-4.01079
H	3.45418	8.15456	-4.91450
C	3.87990	-4.72657	-3.34271
C	5.05759	-5.29943	-3.87925
C	2.70202	-5.49961	-3.23754
C	5.06011	-6.62466	-4.28435
H	5.97056	-4.70088	-3.97905
C	2.70447	-6.82505	-3.64563
H	1.76858	-5.06008	-2.86071
C	3.88453	-7.39650	-4.16493
H	5.97006	-7.08003	-4.69482
H	1.79304	-7.43141	-3.57196
C	-5.90677	-0.53038	-3.46240
C	-6.97837	-1.20099	-4.09556
C	-5.99289	0.85376	-3.19554
C	-8.12229	-0.49963	-4.44546
H	-6.91156	-2.27271	-4.31482
C	-7.13765	1.55524	-3.54710
H	-5.15800	1.39590	-2.73219
C	-8.20799	0.88096	-4.16896
H	-8.96264	-1.01074	-4.93219
H	-7.21412	2.63191	-3.34838
N	-9.41075	1.61565	-4.53418
N	5.68255	6.65491	-5.14014
N	3.88840	-8.78759	-4.58681
O	6.58647	5.82980	-5.23336
O	5.79549	7.80525	-5.55093
O	2.84587	-9.43134	-4.51155
O	4.93370	-9.27712	-5.00410
O	-9.46633	2.81617	-4.28942
O	-10.33010	1.00826	-5.07384

BLYP-D3(0)/def2-SVP-optimized structure of **12** (CPCM acetonitrile):

C	3.46378024650673	2.24550737332251	2.27571210049049
H	2.52468034753144	2.11658608882125	2.84111476711864
C	4.19366323917275	0.89508260564025	1.95984924678836
H	3.54541715390498	0.00324653294634	2.03389199438995
N	4.62357686212824	1.10522398115543	0.56271059675948
N	3.21091536408499	2.81319494200330	0.96308132058765
N	4.48562756738752	2.96241920702866	3.08794634872719
N	5.26140450689892	0.84381281221917	2.93452296781751
C	5.51932055615594	2.07941118677018	3.49962172002551
C	3.92016334794367	2.14097319246591	-0.03746184353841
O	6.44937138031089	2.33873765486628	4.25982331579934

O	3.90595300940674	2.41337954089582	-1.24194451588149
C	6.15806954532521	-0.29395176683310	3.14707155882127
H	7.13368750796828	-0.14558791031841	2.63839635483457
H	6.33941603872230	-0.41370143441528	4.23172022706790
C	0.46556532219195	-2.74449545653519	0.82756391929991
H	0.74209911962058	-1.68999725655278	0.63553814186646
C	-1.07418590877669	-3.02568113285074	0.71405841543574
H	-1.68822742825999	-2.10363153759689	0.67275974829844
N	-1.20203973526667	-3.78999638093435	-0.51542814177669
N	1.02294867064045	-3.62499008792255	-0.21478346386715
N	0.81367344552096	-3.13806097396694	2.19690468089042
N	-1.35590927526119	-3.77632355442241	1.93575611648916
C	-0.29417692410635	-3.81057483017710	2.81357468633075
C	0.03630933926074	-4.19834348937719	-1.01116510036344
O	-0.28233788414708	-4.28146904562008	3.94792244696934
O	0.20494881851755	-4.92806345186638	-1.99113140306612
C	-2.66315315637913	-4.35722789132731	2.22533306839543
H	-2.81822299274919	-5.30768851565738	1.67303126683729
H	-2.72550738581676	-4.55870163506317	3.31017967973050
C	-3.90126285592991	-0.21561549120975	1.93186823186162
H	-3.53001361127014	-1.18586883645273	1.55881976278016
C	-2.75818706239681	0.77392110638947	2.32388839803816
H	-1.79664850680310	0.57328994674031	1.80820730937008
N	-3.33291918480441	2.06624041714471	1.88569853596141
N	-4.64812547117582	0.50273933666152	0.90949695199966
N	-4.59924005580778	-0.37792786019645	3.24044117801347
N	-2.63281393496790	0.61861872063885	3.75238614436265
C	-3.77077516059066	0.06353170857984	4.30952622804413
C	-4.33969414470529	1.87313878746963	0.94132711710199
O	-3.99896762516039	-0.04919443208303	5.51190262956251
O	-4.85270539230826	2.74272491766396	0.23380534055835
C	-1.62012991522435	1.27687931857521	4.58047864733322
H	-2.05793078820255	2.11517483329429	5.16053032737039
H	-1.17380975690282	0.55165897959706	5.28790875131601
C	0.12338179364720	2.55848787656716	-0.47463344278765
H	0.30022044704555	1.46981622872933	-0.41717778986454
C	-1.35207474735194	2.96832079444063	-0.23828136408372
H	-2.07400442906898	2.16275798966468	-0.46329311445176
N	-1.39217736735557	3.31951782454715	1.16552619988922
N	0.80056639510666	3.24276324560841	0.66096357948421
N	0.44987913108530	3.09616686715703	-1.78513537993790
N	-1.50367106594290	4.08911432789418	-1.17943469679466
C	-0.50765615774586	4.12331662792700	-2.13040449581918
C	-0.11159697647227	3.54027164632565	1.67213063840125
O	-0.43470514145880	4.84624187785241	-3.11837053945258
O	0.14902005786561	3.92226591640173	2.81772216356865
C	-2.63784408778883	5.00858857827438	-1.15126939897605
H	-2.51534443848295	5.77021272527047	-0.35282093195941
H	-2.70622219243008	5.51642070155085	-2.13032516810390
C	-3.30357305866841	-0.36421738862582	-1.99767147243600
H	-2.78115259546064	0.49498948814545	-1.54299183553075
C	-2.34187679311265	-1.58837266270573	-2.21011252912810
H	-1.35244138378635	-1.46182037897928	-1.72539687739003
N	-3.07401451905176	-2.69959323820652	-1.59097484202698
N	-4.34099535379575	-0.88501218015480	-1.10795681710579
N	-3.75001028274380	-0.05977196868242	-3.37319773906087
N	-2.18692042955828	-1.67230436913235	-3.64664133231512

C	-3.05134230085735	-0.84645946952125	-4.33498405590162
C	-4.19403985188289	-2.25897782332796	-0.89361873589057
O	-3.16286340391130	-0.77208415524479	-5.55647129389563
O	-4.91463146765346	-2.95926363100813	-0.17700019478836
C	-1.36207670572580	-2.67836496072975	-4.31135153018704
H	-1.92547317259099	-3.61681445074656	-4.49738423832109
H	-1.00722760813853	-2.27739702370237	-5.27829221786480
C	3.11948665518823	-2.18923231362092	-2.25889795325278
H	2.06932501784807	-2.14770391434712	-2.59901771319181
C	3.77897744189535	-0.77411537283965	-2.10089212096086
H	3.05541642433734	0.05860673550555	-2.03965867290722
N	4.51101003746944	-0.92394527327660	-0.82783534436416
N	3.20950958428315	-2.75878786437253	-0.92285466418034
N	3.99839790191003	-2.81945586440572	-3.28093383086810
N	4.61311973448976	-0.64480647224454	-3.27738134906973
C	4.84283042693324	-1.85802260766102	-3.89646780315030
C	4.07160254982294	-2.02260358161258	-0.10336303770475
O	5.60307263382239	-2.05106668707141	-4.84274924973068
O	4.37386602860551	-2.29584731333031	1.06259795663161
C	5.33082786269810	0.56647720079927	-3.67890864524306
H	6.40261945606586	0.52033350459840	-3.39250345954909
H	5.26560570183208	0.67987603585103	-4.77736131043669
C	2.15918938999233	3.76985585920014	0.63433250294763
H	2.37461166196938	4.12796222623061	-0.38945530273609
H	2.19828306704295	4.62784581823925	1.32633913602262
C	-2.61337731771219	3.32997934917507	1.96737484162177
H	-3.31007677101132	4.11693088986630	1.62404533471905
H	-2.31312382209111	3.55114335321773	3.00646763168148
C	-5.26481325862236	-0.10071585100055	-0.28319433368866
H	-5.68672047082140	0.73369183288391	-0.86758964867967
H	-6.08411253495794	-0.77954162039682	0.00650266646750
C	-2.44302441439435	-3.97754766979546	-1.25661135983152
H	-2.18172315056550	-4.54954412963593	-2.16472951482330
H	-3.19056726926480	-4.54846548613287	-0.67620694084522
C	2.44216586347733	-3.89093847699988	-0.40563637574400
H	2.51668658796304	-4.75587665748652	-1.08838705744517
H	2.90278988841751	-4.15245120340169	0.56552645307747
C	5.34026120681308	0.11266707973304	-0.22830967184334
H	5.87666758789094	0.64564813394768	-1.02973080675665
H	6.08703236512862	-0.37990685979698	0.41526233343489
H	5.67239410054484	-1.20017511359528	2.74837922171456
H	-0.82530255759452	1.67900207331014	3.92868499829063
H	-3.46168493448634	-3.64833378115581	1.93119969367512
H	4.85741059252187	1.43134964606207	-3.18586363392243
H	-0.49074782375645	-2.91336682383688	-3.67308230963917
H	-3.56874344953962	4.44346750034176	-0.95660994324551
C	-5.83969495486948	-1.01652714838605	3.42029703988999
C	-6.68589709489468	-0.66265389578460	4.51110513987546
C	-6.27371733089243	-2.01115225210017	2.50483334026226
C	-7.92120966688732	-1.29452823853842	4.68056862418005
H	-6.37214518616802	0.11077969486354	5.21876170132880
C	-7.51572604802232	-2.63454611893579	2.66717206589019
H	-5.64567701459397	-2.32102310726373	1.66247804769285
C	-8.33366426818453	-2.27726533566925	3.75675597335217
H	-8.58023547272517	-1.02206429738097	5.51334740735023
H	-7.84865013140276	-3.40539499896230	1.96225593797492
C	1.56280550069560	-2.25820889839183	3.05583294202240

C	2.73512667739875	-2.71727361979187	3.68813704886722
C	1.07478436166168	-0.95516369953510	3.31831039432792
C	3.44390028291143	-1.87097378608428	4.55479754056878
H	3.09553208859913	-3.73143677527151	3.47868246967085
C	1.77055326030674	-0.10337797101898	4.18391283246428
H	0.13643567733389	-0.62298175569005	2.85550107745109
C	2.96257873019143	-0.56648427300602	4.77220415821987
H	4.37098337345668	-2.19897788889658	5.03771799694840
H	1.40389532953581	0.90229233108501	4.41320555928301
C	4.31009096029259	4.24744575408587	3.63597999581701
C	5.43772509526718	5.06222215936935	3.94185684543612
C	3.00599557301179	4.75236455993914	3.88211890998354
C	5.26078588661841	6.33761294780058	4.48657349689109
H	6.44824385467477	4.69151040621657	3.74492264885314
C	2.82791985085033	6.03384453442009	4.41361025629847
H	2.11365389764861	4.15816018119397	3.65850374232479
C	3.95613182181743	6.82025839989805	4.71913646368441
H	6.12376400711967	6.97308093710285	4.71769428446398
H	1.82066225915809	6.42342438054645	4.60227001969760
N	-9.64037079585326	-2.93738356857260	3.93264458478141
O	-9.99669985753357	-3.77785057853261	3.08358418060433
O	-10.33117204305403	-2.62648218746117	4.92268217354969
N	3.75164373776450	0.37001374974245	5.60950722442360
O	3.38518039809023	1.55999964829493	5.65889855396712
O	4.75222271599606	-0.06886366659143	6.20178306289500
N	3.76998122330506	8.16958807714276	5.28357165213348
O	2.60643657885351	8.58932073034680	5.43977985385134
O	4.78385971244495	8.83145588933848	5.58052691030596
C	0.97143123939144	2.26801488208197	-2.82352746689004
C	0.54393986161628	0.92113321231277	-2.94498499706606
C	1.88182308191179	2.80164729955098	-3.76420296496031
C	1.06584727374274	0.09959334483491	-3.94739134488357
H	-0.20775012376428	0.51324771983623	-2.26018623555491
C	2.41368729147992	1.98301698951366	-4.76979069733573
H	2.19504550505705	3.84798466776829	-3.67300368180004
C	2.02281382730915	0.63020472647104	-4.83202490362406
H	0.74595323598085	-0.94092329495784	-4.04833653876945
H	3.15387240579350	2.36959624278422	-5.47926005408406
C	3.80914980894688	-4.10690795065297	-3.82349870006478
C	4.92189409223103	-4.85808405465216	-4.29533839168581
C	2.51120206029469	-4.66774599085184	-3.90090527790868
C	4.73194120249653	-6.13496687286798	-4.83285998615267
H	5.93036335067637	-4.43676138262617	-4.23002653952940
C	2.31879337795146	-5.95252975632033	-4.41735162284799
H	1.63928829346636	-4.11185040892298	-3.54971272466461
C	3.43102031398720	-6.67840686990953	-4.88679589568238
H	5.58255583645972	-6.72581734249692	-5.19218545023110
H	1.31500497419549	-6.38971096580063	-4.46206009958298
C	-4.57749333967489	1.02924729435112	-3.71743979053118
C	-5.40869321642319	0.97018078051470	-4.87201030422349
C	-4.60016847241184	2.19924956780962	-2.91348095715315
C	-6.22167439523887	2.05556109706685	-5.21369850249926
H	-5.41596363534751	0.06965773732509	-5.49354749455904
C	-5.42098990628513	3.28068868652920	-3.24585111311960
H	-3.97605188888278	2.28284966553326	-2.02153489733957
C	-6.22548926470652	3.20621551898101	-4.39890203752379
H	-6.86898035224582	2.01374232195597	-6.09759967107512

H	-5.43244677608520	4.18350848512001	-2.62501860316675
N	-7.08930611483829	4.34843709249500	-4.75487885856034
N	2.66298630949790	-0.27422095902641	-5.80910329585981
N	3.23301968470412	-8.03341468188791	-5.43683359659540
O	2.34987292682976	-1.48153964964265	-5.78751999834533
O	3.50453634040838	0.20288816642126	-6.59267194383074
O	2.08199963831768	-8.51157554925954	-5.42832373190112
O	4.22506967749730	-8.63969801003887	-5.88623906077368
O	-7.10148046350994	5.33736914859635	-3.99644408787265
O	-7.76709548079929	4.27319683649511	-5.79772606802910
